# In Situ Construction of Cu^1+^/Cu^0^ and Cu^2+^/Cu^0^ Pairs of Cu‐Based Catalysts for Electrocatalytic Nitrate Reduction

**DOI:** 10.1002/advs.202517773

**Published:** 2025-11-29

**Authors:** Shanna An, Jiali Ren, Yanjun Xue, Jian Tian

**Affiliations:** ^1^ School of Materials Science and Engineering Shandong University of Science and Technology Qingdao 266590 China

**Keywords:** Cu species reconstruction, Cu^1+^/Cu^0^ pairs, Cu^2+^/Cu^0^ pairs, in situ spectra, NH_3_ electrosynthesis

## Abstract

Electrocatalytic nitrate reduction reaction (NO_3_RR) as a sustainable nitrogen cycle regulation strategy provides a new pathway to achieve carbon neutrality goals. In this study, CuO, Cu_2_P_2_O_7_ and Cu_3_(PO_4_)_2_ (CuPO) are synthesized as pre‐catalysts via a sol‐gel process for NO_3_RR, in which CuO exhibits excellent NH_3_ yields of 8.16 mg h^−1^ mg_cat_^−1^ (FE = 95.72%, ‐0.95 V vs. RHE) compared to Cu_2_P_2_O_7_ (7.33 mg h^−1^mg_cat_^−1^, 94.88%) and Cu_3_(PO_4_)_2_ (6.53 mg h^−1^mg_cat_^−1^, 92.04%). The combination of in situ Raman and XPS spectra reveal that the pre‐catalyst surface is reconfigured to form stable active sites of Cu^1+^/Cu^0^ (CuO‐derived) and Cu^2+^/Cu^0^ pairs (CuPO‐derived) during the NO_3_RR process. Tracking the evolution of intermediates using online differential electrochemical mass spectrometry (DEMS) spectra and in situ Fourier transform infrared spectroscopy (FT‐IR) spectra reveal that Cu^1+^/Cu^0^ pairs possess rapid catalytic kinetics for the conversion of *NO_3_^−^ to *NO_2_^−^. Density functional theory (DFT) calculations confirm that Cu^1+^/Cu^0^ exhibits a lower potential‐determining step, and its exceptional *H generation and enrichment capabilities promote further hydrogenation reactions, thereby achieving excellent activity and selectivity in NH_3_ production via NO_3_RR. This study reveals the distinct advantages of reconstructed active sites in Cu‐based catalysts during NO_3_RR, providing guidance for designing other advanced catalysts.

## Graphical abstract

CuO and CuPO (Cu_2_P_2_O_7_ and Cu_3_(PO_4_)_2_) as precursors are in suit construction two distinct copper‐based active sites during the NO_3_RR reaction: Cu^1+^/Cu^0^ (derived from CuO) and Cu^2+^/Cu^0^ (derived from CuPO). Cu^1+^/Cu^0^ is the key to achieving high activity and high selectivity. Combining online DEMS and in situ FT‐IR spectroscopy with DFT calculations, the reaction mechanism of Cu^1+^/Cu^0^ is confirmed.

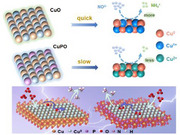

## 1 Introduction

Ammonia (NH_3_) occupies a unique position at the nexus of global food security and sustainable energy transition.^[^ ^1^, ^2^ ^]^ However, NH_3_ is mainly produced by Haber‐Bosch process, which operates under energy‐intensive conditions (400–500 °C, 15–25 MPa) and consumes 1–2% of global energy, along with the high emission of CO_2_ gas.^[^ ^3^, ^4^, ^5^ ^]^ This environmental‐economic paradox has spurred intensive exploration of the electrochemical routes to NH_3_ synthesis. In parallel, the nitrate reduction reaction (NO_3_RR) has emerged as a promising pathway for sustainable NH_3_ production from nitrate waste streams, presenting a complementary strategy to conventional nitrogen management. The N─O bond dissociation energy (204 kJ mol^−1^) in nitrate is significantly lower than that of the N≡N bond (941 kJ mol^−1^) in nitrogen, and NO_3_RR overcomes the mass‐transfer limitation associated with the low solubility of N_2_ (0.6 mM at 298 K) in nitrogen reduction reaction (NRR) systems.^[^ ^6^, ^7^, ^8^ ^]^ Furthermore, NO_3_RR enables the valorization of nitrate contaminants under ambient conditions, simultaneously addressing environmental remediation and sustainable NH_3_ production.^[^ ^9^ ^]^

NO_3_RR involves multiple proton/electron transfer and highly complex reaction pathways with multiple intermediates, and the rational design of catalysts is crucial to modulate the selectivity toward NH_3_ product.^[^ ^10^, ^11^ ^]^ Copper‐based catalysts are widely regarded as ideal candidate for NO_3_RR due to the energy level matching properties of Cu d‐orbitals and the lowest unoccupied molecular π* orbitals (LUMO) of nitrate.^[^ ^12^, ^13^ ^]^ To date, researchers have investigated a large number of Cu‐based catalysts and observed that Cu species tend to undergo reconfiguration to serve as the active sites for the NO_3_RR process.^[^ ^14^, ^15^, ^16^, ^17^ ^]^ For example, Wang et al.^[^ ^18^ ^]^ reported that CuO nanowire arrays in situ reconfigured during electrochemical reduction to a mixed phase of Cu/Cu_2_O as the active phase of the NO_3_RR. Zheng et al.^[^ ^14^ ^]^ reported CuO nanoparticles incorporated on nitrogen‐doped porous carbon (CuO@NC) catalyst, where Cu species were reconfigured to construct Cu(I)‐Cu(II) pairs as active sites during NO_3_RR. However, clarifying the relative advantages of different active sites after reconstruction and establishing design strategies for efficient catalysts based on this remains a critical issue to be urgently addressed.

Recently, researchers have found through theoretical studies that copper phosphates (e.g., cupric pyrophosphate Cu_2_P_2_O_7_^[^ ^19^ ^]^ and cupric orthophosphate Cu_3_(PO_4_)_2_, denoted as CuPO)^[^ ^20^ ^]^ can preserve Cu^2+^ sites in their material structure design under electrochemical reduction conditions.^[^ ^21^ ^]^ Inspired by these findings, it is feasible to design the electrochemical reconstruction of Cu^2+^‐based precursors to form stable active sites containing copper species in different valence states for NO_3_RR. Such an approach would also enable comparative studies to elucidate the relative advantages and mechanistic roles of distinct active sites.

**Scheme 1:**
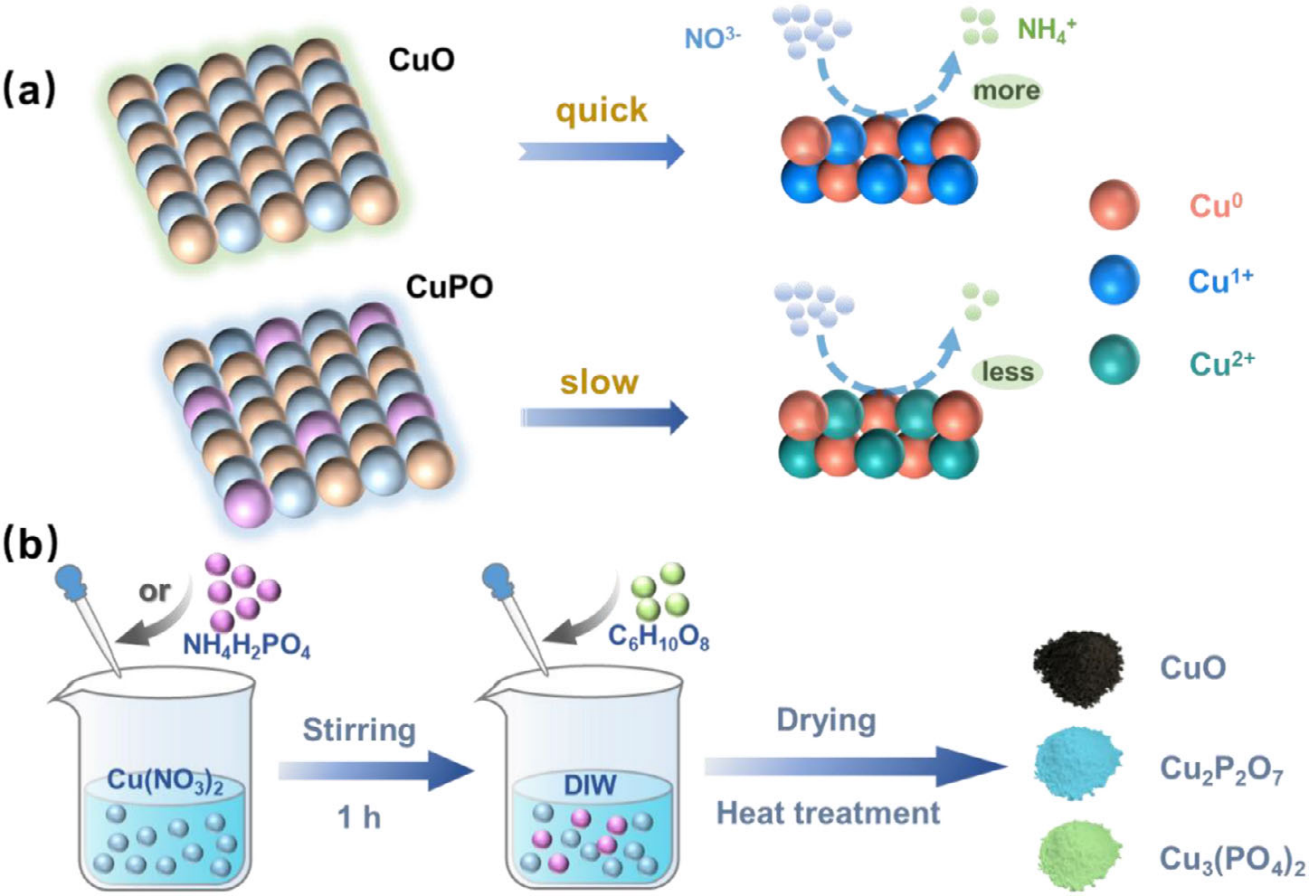
Schematic representation of a) electrochemical reconstruction on CuO and CuPO and b) preparation process of CuO, Cu_2_P_2_O_7_, and Cu_3_(PO_4_)_2_.

In our work, CuO and Cu‐based phosphates (CuPO) were used as effective precatalysts for electrocatalytic NO_3_RR, as their electrochemical reconstruction can generate active Cu species with tunable valence states for NO_3_^−^ conversion, which CuO exhibits excellent NH_3_ yields of 8.16 mg h^−1^ mg_cat_^−1^ (FE = 95.72%, ‐0.95 V vs. RHE) compared to Cu_2_P_2_O_7_ (7.33 mg h^−1^mg_cat_^−1^, 94.88%) and Cu_3_(PO_4_)_2_ (6.53 mg h^−1^mg_cat_^−1^, 92.04%). In situ Raman and XPS spectra reveal that the electrochemical reconstruction of Cu species results in distinct active sites: Cu^1+^/Cu^0^ (CuO‐derived) and Cu^2+^/Cu^0^ pairs (CuPO‐derived) (**Scheme** **1**a). Operando differential electrochemical mass spectrometry and in situ Fourier transform infrared spectroscopy reveal that the Cu^1+^/Cu^0^ active pairs possess rapid catalytic kinetics for the conversion of *NO_3_^−^ to *NO_2_^−^. Integrating the Density functional theory (DFT) calculation results demonstrate the superior activity of Cu^1+^/Cu^0^ pairs, which stems from their ability to lower the adsorption and activation energy barriers of intermediates and generate active hydrogen (*H) more efficiently, thereby accelerating the reaction kinetics. The work elucidates the constitutive relationship between the dynamic reconfiguration mechanism of the active phase and the NO_3_RR activity, and provides a key theoretical basis for the rational design of efficient nitrate‐ammonia electrocatalysts.

## 2 Results and Discussion

### 2.1 Catalysts Synthesis and Structure Investigation

**Figure 1:**
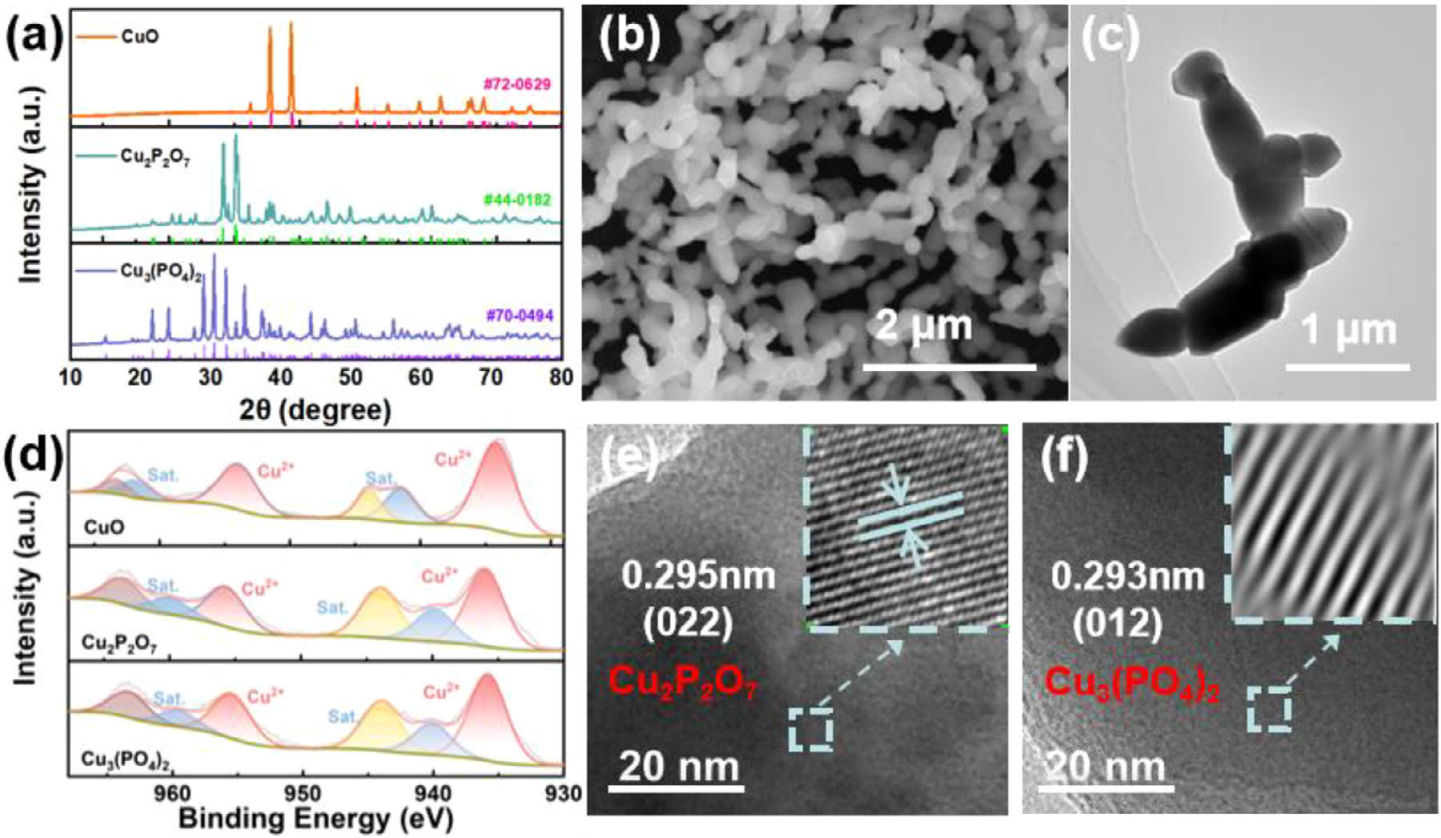
a) XRD patterns of CuO, Cu_2_P_2_O_7_ and Cu_3_(PO_4_)_2_; b) SEM image of CuO; c) TEM image of CuO; d) Cu 2p XPS spectra of CuO, Cu_2_P_2_O_7_ and Cu_3_(PO_4_)_2_; HRTEM images of e) Cu_2_P_2_O_7_ and (f) Cu_3_(PO_4_)_2_.

A simple and scalable sol‐gel method was developed for the synthesis of CuO and CuPO (copper phosphate, Cu_2_P_2_O_7_ and Cu_3_(PO_4_)_2_), where CuPO was prepared by introducing phosphate salts into the CuO synthesis system (Scheme 1b, details in Supporting Information). The XRD patterns of the catalyst powders are shown in **Figure** **1**a. The diffraction peaks are in good agreement with the standard crystal structures of CuO (PDF#72‐0629), Cu_2_P_2_O_7_ (PDF#44‐0182) and Cu_3_(PO_4_)_2_ (PDF#70‐0494), with no impurity signals detected. The morphological and structural characteristics of the catalysts are observed via scanning electron microscopy (SEM) and transmission electron microscopy (TEM) images. All three Cu‐based catalysts exhibit similar morphological features of nanoparticle‐assembled networks, which are conducive to electrolyte adsorption in NO_3_RR. SEM‐EDS mapping images demonstrated the spatial homogeneity of Cu, P, and O elemental distributions, further confirming the successful preparation of the Cu‐based pre‐catalysts (Figure 1b,c; Figure S1, Supporting Information). To investigate the valence state of Cu in the catalysts, X‐ray photoelectron spectroscopy (XPS) characterization is performed on CuO and CuPO (comprising Cu_2_P_2_O_7_ and Cu_3_(PO_4_)_2_). The survey spectra indicate the presence of both Cu and O elements in all samples, whereas the P element is specifically detected in the CuPO samples (Figure S2, Supporting Information). The Cu 2p spectra show that all three Cu‐based catalysts exhibit characteristic signals of Cu^2+^ species (Figure 1d). Furthermore, the high‐resolution transmission electron microscopy (HRTEM) images of CuPO (Figure 1e,f) revealed distinct lattice fringes. The fringe spacing of 0.295 nm corresponds to the (022) plane of the monoclinic phase Cu_2_P_2_O_7_, whereas the spacing of 0.293 nm is associated with the (012) plane of the triclinic phase Cu_3_(PO_4_)_2_, confirming the successful synthesis of the pre‐catalysts.

### 2.2 Electrochemical NO_3_^−^ Conversion

**Figure 2:**
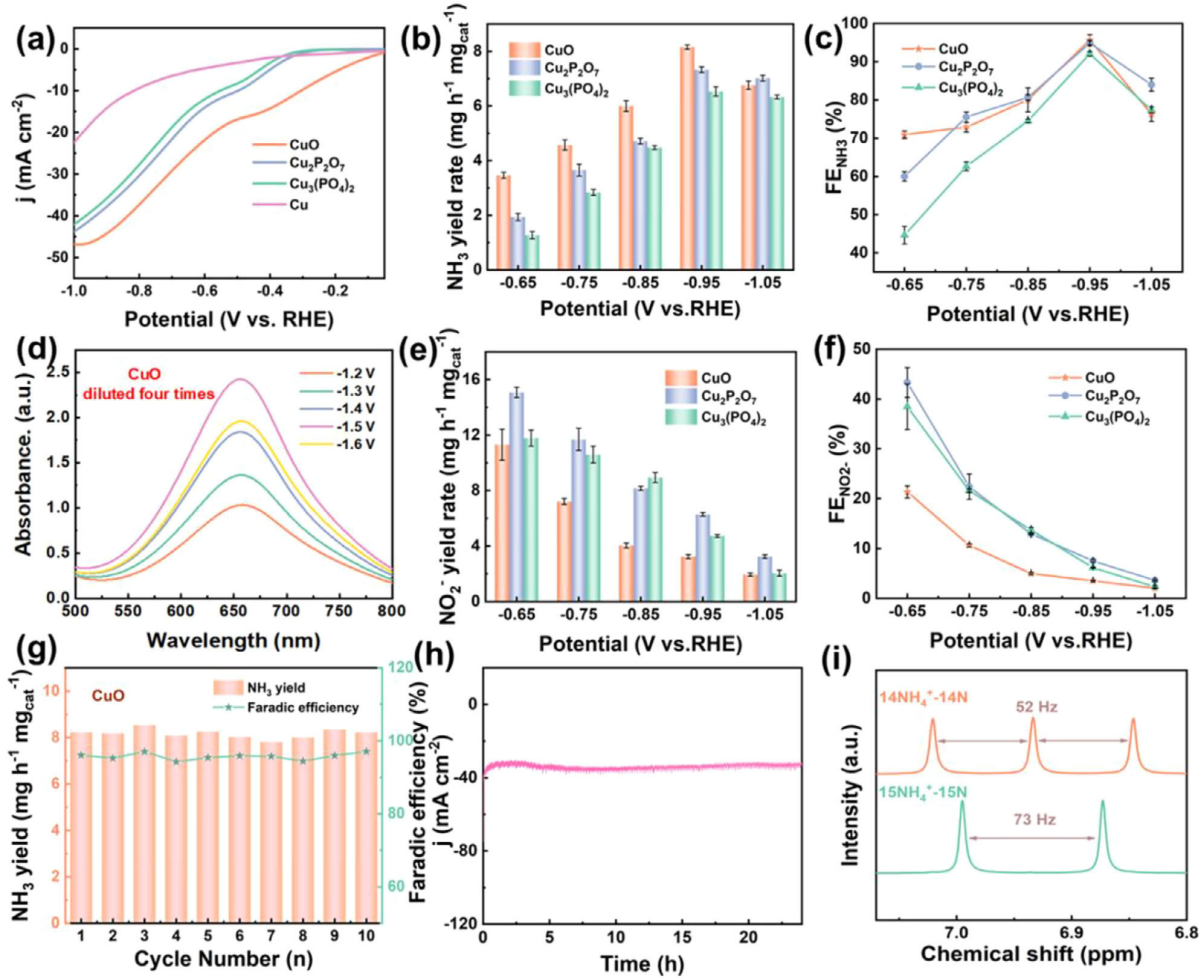
a) LSV curves of Cu, CuO and CuPO in 0.1 M N_2_SO_4_ with 0.1 M KNO_3_; b) NH_3_ yield rate and c) Faradaic efficiency of CuO and CuPO at different potentials; d) UV–vis absorption spectra of electrolyte after CuO NO_3_RR; e) NO_2_^−^ yield rate and f) Faradaic efficiency of CuO and CuPO at different potentials; g) Cycling stability of CuO at ‐0.95 V versus RHE; h) 24 h I‐t curves of CuO; i) ^1^H NMR spectra of the electrolyte after NO_3_RR using ^15^NO_3_^−^ and ^14^NO_3_^−^ as the nitrogen source, respectively. Error bars represent the standard deviation of at least three independent measurements, with the center value being their average.

The NO_3_RR performance of CuO and CuPO catalysts is measured in an H‐type electrolytic cell in a static three‐electrode system under ambient conditions. All potentials are referenced to RHE. First, LSV curves are tested in 0.1 M Na_2_SO_4_ electrolyte solutions containing or not 0.1 M KNO_3_ (Figure S3, Supporting Information). With the addition of 0.1 M KNO_3_, the current density at the same potential is significantly enhanced, indicating that NO_3_RR is preferred over HER on the catalyst. Furthermore, the current density of the LSV curve of CuO is higher than that of CuPO (**Figure** **2**a). The LSV curves of pure Cu^0^ are also compared, revealing that at the same test potential, the current density of pure Cu^0^ is significantly lower than that of the three catalysts (CuO, Cu_2_P_2_O7, and Cu_3_(PO_4_)_2_). According to the LSV curves, two distinct reduction peaks can be observed: the first corresponds to the reduction of NO_3_^−^ to NO_2_^−^,^[^ ^22^, ^23^ ^]^ during which CuO exhibits the lowest onset potential; the second corresponds to the further reduction of NO_2_^−^ to generate NH_3_, where CuO demonstrates faster kinetics and thus produces NH_3_ more rapidly. Ammonia yield and selectivity are critical parameters for evaluating the performance of NO_3_RR. Figure 2b,c demonstrate the average NH_3_ yield and Faraday efficiency with a selected potential window of −0.65 to −1.05 V based on LSV. The ammonia production rates and Faraday efficiencies of CuO and CuPO show a good volcano‐like trend. Notably, CuO achieves the highest NH_3_ yield of 8.16 mg h^−1^mg_cat_^−1^ at −0.95 V, surpassing the performance of Cu_2_P_2_O_7_ (7.33 mg h^−1^mg_cat_^−1^) and Cu_3_(PO_4_)_2_ (6.53 mg h^−1^mg_cat_^−1^). All three catalysts attain their maximum Faradaic Efficiency (FE) at ‐0.95 V, with CuO (95.72%), Cu_2_P_2_O_7_ (94.88%), and Cu_3_(PO_4_)_2_ (92.04%) exhibiting comparable FE values exceeding 90%. The concentration of NH_4_^+^, NO_2_^−^, NO_3_^−^ and N_2_H_4_ are calculated from the standard curve (Figures S4 and S5, Supporting Information). Figure S6 (Supporting Information) demonstrates the time‐varying test of CuO at different potentials in an electrolyte containing 0.1 M KNO_3_. The corresponding absorption curves obtained according to the staining method are displayed in Figure 2d and Figure S7 (Supporting Information). NO_2_^−^ is identified as the main intermediate by‐product during the NO_3_RR process. Colorimetric detection of NO_2_^−^ in the electrolyte (Figure 2e,f) reveals that both the yield and FE of NO_2_^−^ are lower for CuO compared to CuPO. This observation indicates that CuO possesses faster conversion kinetics toward NO_2_^−^, which is favorable for the selective synthesis of NH_3._ Other possible by‐products including N_2_H_4_⋅H_2_O are also analyzed, as shown in Figure S8 (Supporting Information), N_2_H_4_⋅H_2_O is barely detected after electrolysis by NO_3_RR. This further confirms the better selectivity of CuO for ammonia production.

The durability of catalyst materials is also another key indicator in ammonia electrosynthesis. Therefore, we explored the durability of CuO by cycling tests at the optimal potential of ‐0.95 V (Figure 2g). The test results indicate that after 10 cycles of testing, the ammonia yield and FE value remain essentially stable, demonstrating excellent cyclic stability. During a 24 h long‐term stability test, CuO exhibits slightly excellent stability (Figure 2h). Extended stability testing was conducted, as shown in Figure S9 (Supporting Information). After 95 hours of continuous testing, a slight decay in current density is observed, demonstrating good long‐term stability. Despite the simplicity of the prepared catalysts, the electrocatalysts prepared in this paper still show excellent NO_3_RR performance compared to other Cu‐based catalysts reported in the literature (Table S1, Supporting Information).

We have performed a series of controlled experiments to elucidate the origin of ammonia. Figure S10 (Supporting Information) shows negligible ammonia production at a blank electrode, at open‐circuit potential, and under pure 0.1 M Na_2_SO_4_ conditions without KNO_3_, suggesting that all of the NH_3_ produced is from electrocatalysis. When the N source is ^15^N‐NO_3_, a double peak coupling corresponding to ^15^NH_4_^+^ is observed from the ^1^H nuclear magnetic resonance (NMR) spectrum (Figure 2i), and a triple peak coupling corresponding to ^14^NH_4_^+^ is demonstrated when the N source was ^14^N‐NO_3_^−^. Collectively, these results confirm that the produced NH_3_ originates from the electrocatalytic conversion of NO_3_‐N. Importantly, the quantitative ammonia yield obtained by integrating the peak areas of the 1H NMR spectrum is comparable to the results from the staining method (Figure S11, Supporting Information), confirming the accuracy of the staining method for measuring ammonia production rates.

### 2.3 In Situ Electrocatalytic Reconstruction

In situ structural reconstruction of the catalysts is observed following NO_3_RR. For clarity, the reconstructed catalysts after NO_3_RR are denoted as CuO_re._, Cu_2_P_2_O_7re._ and Cu_3_(PO_4_)_2re._, respectively. To systematically study the structural and compositional evolution of the catalyst after electrolysis, these materials are carefully transferred to a carbon cloth substrate to serve as the working electrode for the electrocatalytic reaction process, ensuring consistency between the reaction environment and subsequent characterization. XRD and XPS analyses are then performed. XRD measurements (Figure S12, Supporting Information) reveal a critical structural transformation associated with the in situ reconstruction: as NO_3_RR proceeded, all three catalyst systems exhibit emerging diffraction peaks at 2θ values corresponding to metallic Cu (PDF#04‐0836). Comparison of electrolysis durations of 0.5 h and 2 h revealed a slight increase in the intensity of the diffraction peaks, indicating that initial copper oxide‐based structures transformed into metallic Cu within a relatively short time frame. This observation provides direct crystallographic evidence for the reductive transformation of copper species during the in situ reconstruction process.

**Figure 3:**
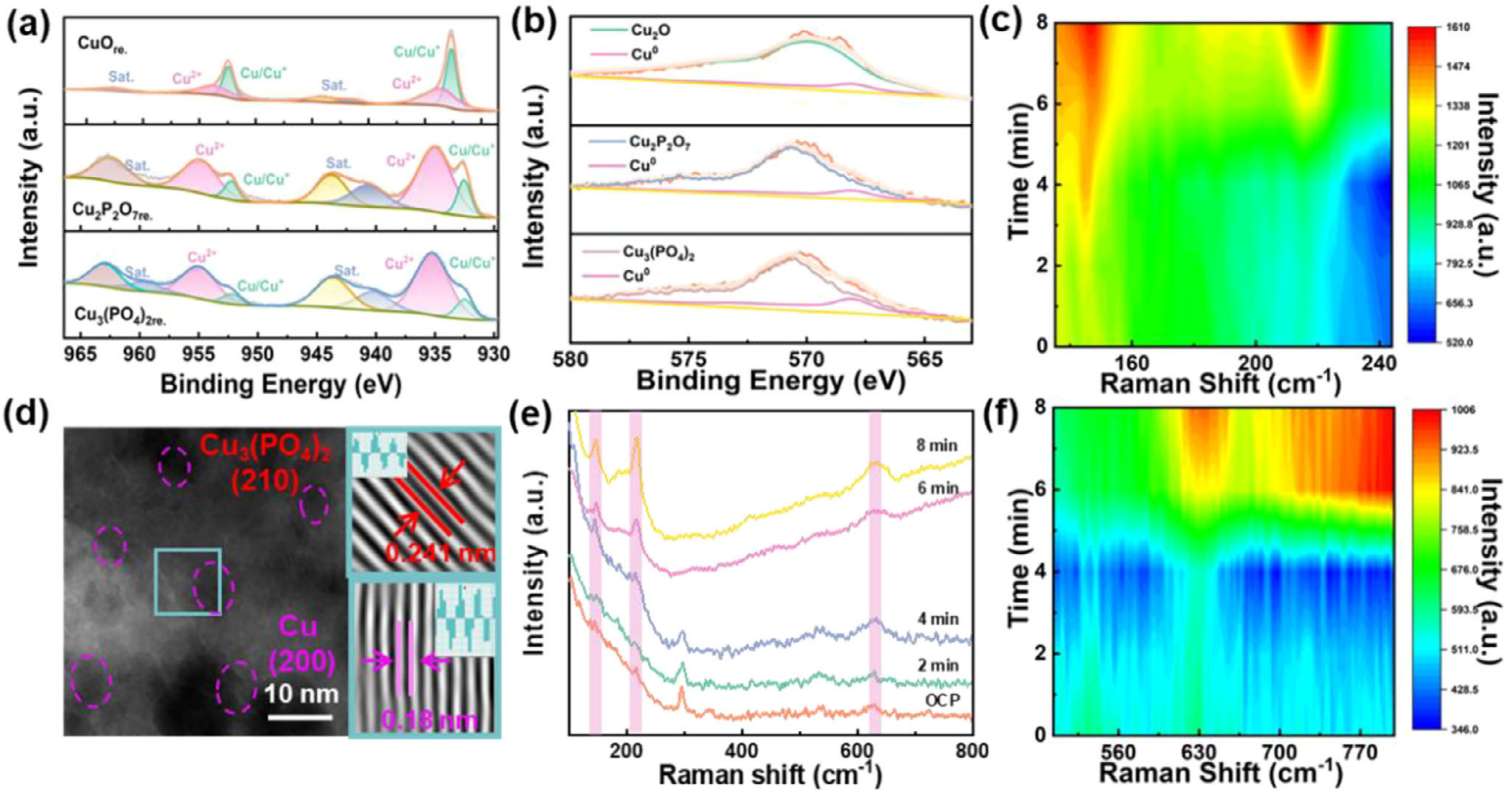
a) Cu 2p XPS spectra of CuO_re._ and CuPO_re._ after 1 h of NO_3_RR; b) Cu LMM AES spectra of CuO_re_ and CuPO_re_ after 1 h of NO_3_RR; c) In situ electrochemical Raman contour map of 135–244 cm^−1^; d) HRTEM images of Cu_3_(PO_4_)_2re._ after 1 h of NO_3_RR; e) In situ electrochemical Raman spectra of CuO under given potential with different time Raman profiles; f) In situ electrochemical Raman contour map of 503–800 cm^−1^.

To gain deeper insights into the oxidation states of Cu species within the in situ reconstructed catalysts, XPS and AES (Auger Electron Spectroscopy) characterizations are performed. In the Cu 2p XPS spectrum of CuO_re._ (**Figure** **3**a), the dominant Cu species are characterized by well‐defined peaks that are predominantly associated with Cu^1+^ or Cu^0^, consistent with the characteristic binding energies reported for these lower oxidation states in the literature.^[^ ^24^, ^25^ ^]^ This observation indicates the preferential formation of reduced Cu species in CuO_re._ following the reconstruction process. By sharp contrast, the Cu 2p spectral features of CuPO_re._ reveal that Cu^2+^ remains the dominant oxidation state after in situ reconstruction, with faint contributions attributable to Cu^1+^ or Cu^0^ as minor components. We measured LMM spectra of the precursors Cu_2_P_2_O_7_ and Cu_3_(PO_4_)_2_ as reference spectra (Figure S13, Supporting Information), then acquired Cu LMM AES spectra (Figure 3b) of the samples to distinguish between Cu^1+^ and Cu^0^ signals. In the CuO_re._ catalyst, characteristic peaks assigned to Cu_2_O (Cu^1+^) and Cu^0^ are observed, confirming that CuO undergoes in situ reconstruction to form Cu^1+^/Cu^0^ under electrochemical NO_3_RR conditions. Meanwhile, the detection of characteristic peaks associated with Cu^0^ of Cu_2_P_2_O_7re._ and Cu_3_(PO_4_)_2re._ demonstrates that the phosphate precursors are partially reduced under electrochemical conditions and lead to their reconstruction into active sites comprising a mixture of Cu^2+^ and Cu^0^,^[^ ^26^ ^]^ in agreement with the findings from the XPS characterization. Based on these comprehensive results, possible hypotheses regarding the dynamic evolution process of CuO and CuPO under electrochemical conditions in the NO_3_RR are obtained. Specifically, CuO_re._ undergoes rapid reductive transformation and structural reconstruction upon exposure to the NO_3_RR environment, leading to the formation of Cu^1+^/Cu^0^ pairs as the primary surface species. In contrast, CuPO_re._ retains Cu^2+^ as the dominant oxidation state after in situ reconstruction, producing only trace amounts of Cu^0^, thus forming Cu^2+^/Cu^0^ pairs as active sites for the reaction. Different structural evolutions are expected to play distinct roles in regulating the catalytic activity and selectivity of the material towards the NO_3_RR reaction.

HRTEM analysis of Cu_3_(PO_4_)_2re._ (Figure 3d) further reveals structural evolutions: within the dominant lattice fringes corresponding to Cu_3_(PO_4_)_2_, additional lattice spacings with smaller d‐values are observed, which could be indexed to the (200) plane of metallic Cu, confirming the in situ formation of Cu^0^ species. Meanwhile, the SAED pattern (Figure S14, Supporting Information) exhibits a diffraction ring corresponding to a lattice spacing of 0.183 nm, which is assigned to the (200) crystal plane of Cu^0^. The SAED patterns confirm the presence of metallic Cu. Complementary TEM‐EDS mapping (Figure S15, Supporting Information) reveals a marginally broader spatial distribution of Cu relative to P and O after NO_3_RR, which is attributed to the partial reduction of Cu^2+^ to Cu^0^ and supporting the in situ formation of Cu^2+^/Cu^0^ structure.

To gain atomic‐level insights into the dynamic reconstruction of Cu species under working conditions, in situ Raman electrochemical measurements are performed on CuO. Under open‐circuit potential (OCP), the Raman spectrum of CuO (Figure 3e) exhibited three characteristic peaks at 298, 334, and 625 cm^−1^, which are well‐documented as the vibrational modes of CuO. Time‐resolved Raman spectra collected during chronoamperometric tests at a constant potential of ‐0.95 V versus RHE exhibit a systematic temporal evolution: vibrational peaks at 148, 218, and 635 cm^−1^, which correspond to the characteristic Raman bands of Cu_2_O,^[^ ^22^ ^]^ emerged gradually with increasing intensity, while the CuO‐specific vibrational features show a concurrent attenuation over the same time scale. This spectral evolution directly indicates the electrochemical transformation of CuO to Cu_2_O. The 2D Raman contour plots (Figure 3c,f) further visualized this temporal evolution, with the increasing intensity of Cu_2_O signals corroborating the progressive reconstruction. Notably, metallic Cu does not exhibit Raman‐active modes, precluding its direct detection via this technique.

Raman spectra of CuPO and CuPO_re_ are shown in Figure S16 (Supporting Information). The peaks in the 1250–950 cm^−1^ region are assigned to P‐O stretching modes of [P_2_O_7_]^4−^,^[^ ^27^ ^]^ while vibrations above 400 cm^−1^ correspond to internal modes of [PO_4_]^3−^ tetrahedron.^[^ ^21^ ^]^ These phosphate‐related features remain intact before and after reconstruction, indicating the stability of the phosphorus‐oxygen framework during NO_3_RR. Within phosphate groups ([P_2_O_7_],^4−^ [PO_4_]^3−^), phosphorus (P) and oxygen (O) form high‐bond‐energy P‐O covalent bonds that firmly anchor oxygen atoms within the phosphate backbone. Phosphate groups play a crucial role in stabilizing oxygen atoms,^[^ ^21^ ^]^ thereby limiting the reduction of Cu^2+^. Collectively, these microscopic and spectroscopic results consolidate our previous findings: CuO undergoes electrochemical reconstruction into a composite of metallic Cu and Cu_2_O, generating Cu^1+^/Cu^0^ pairs as the active sites for NO_3_RR. In contrast, the active sites in CuPO are identified as Cu^2+^/Cu^0^ pairs, derived from the partial reduction of Cu^2+^ during in situ reconstruction while maintaining the integrity of the phosphate matrix. Such distinct differences in the pairs of Cu species as active sites between the different Cu‐catalyst systems, which may directly govern their catalytic performance toward NO_3_RR.

### 2.4 Mechanism Analysis

**Figure 4:**
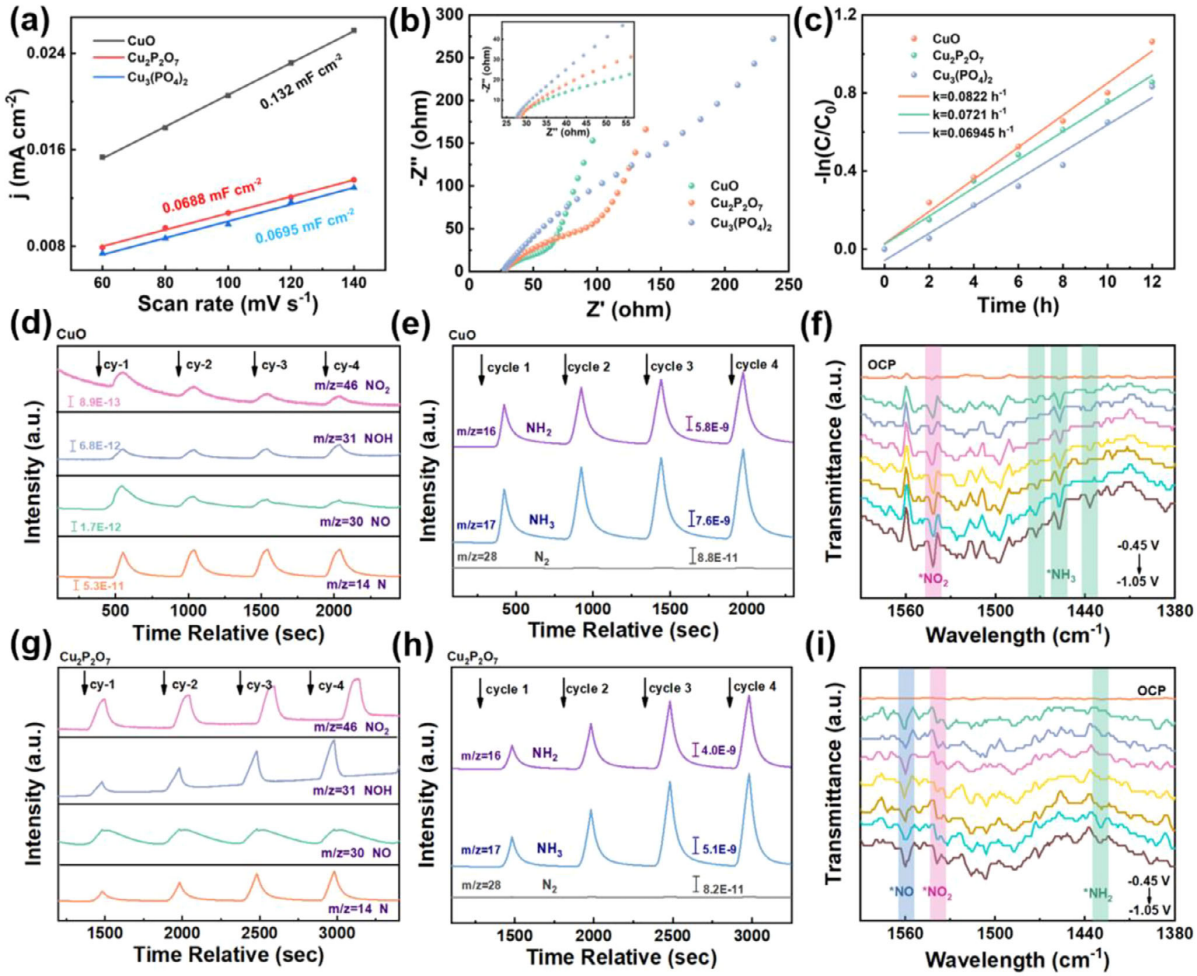
a) The fitted linear relationship between current density and scan rate in CV curves for as‐studied catalysts; b) Electrochemical impedance spectroscopy of catalysts; c) Linearized pseudo‐first‐order kinetics profiles of different catalysts; Electrochemical online DEMS results for the electrocatalytic NO_3_RR over d, e) CuO_re._ and g, h) Cu_2_P_2_O_7re._; In situ FTIR spectra of (f) CuO_re._ and i) Cu_2_P_2_O_7re._ at different potentials for the NO_3_RR process.

To investigate the reason for the high activity of Cu^1+^/Cu^0^ (CuO‐derived), electrochemical tests are conducted on CuO and CuPO. Firstly, the electrochemically active surface areas (ECSAs) are measured to evaluate the intrinsic catalytic performance of the nanomaterials via a series of CV curves are recorded for the three catalysts within the non‐Faraday region. In the CV curves (Figure S17, Supporting Information), the current density gradually increases with the scan rate rising from 60 mV^−1^ to 140 mV^−1^. As illustrated in **Figure** **4**a, CuO exhibits a larger double‐layer capacitance (C_dl_) than CuPO, which is accounted for by its rapid and extensive reconstruction into Cu^1+^/Cu^0^ pairs, resulting in a rougher electrode surface. An increase in ECSA values indicates an increase in the number of catalytic active sites and higher overall ammonia production activity. Interestingly, after calculating the ECSA based on C_dl_ (specific details in Supporting Information) and normalizing ammonia yield accordingly (Figure S18, Supporting Information), a reversal in the activity trend is observed. This discrepancy can be reasonably explained as follows: although the ECSA value of the Cu^1+^/Cu° catalyst is nearly twice that of the Cu^2+^/Cu^0^ sample, the difference in mass‐normalized NH_3_ production rates between the two is less than twofold. This has led to the phenomenon that appears contradictory but is actually reasonable. Furthermore, in CuPO‐derived catalysts, phosphate groups inhibit the deep reduction of Cu^2+^, leading to constrained surface reconstruction and reduced effective surface area. This results in higher surface area‐normalized ammonia yield. Nevertheless, the Cu^1+^/Cu° catalyst retains higher overall activity density. Electrochemical impedance spectroscopy (EIS) shows that CuO has a small charge transfer resistance, suggesting faster charge transfer kinetics at the electrode‐electrolyte interface and thereby enhancing the reduction kinetics of NO_3_RR (Figure 4b). To further study the apparent kinetic activity of the three catalyst systems, the apparent rate constants (K_ap_) for NO_3_^−^ conversion are determined via 12‐h chronoamperometric measurements at their respective electrode interfaces (Figure 4c; Figure S19, Supporting Information).^[^ ^28^ ^]^ Kinetic analysis reveals that the residual nitrate concentration in the electrolyte decreases most significantly over time at the CuO electrode. Calculation of the K_ap_ values further confirm a distinct reactivity hierarchy among the catalysts, with the NO_3_^−^ conversion rates following the order: K_ap_ (CuO) > K_ap_ (Cu_2_P_2_O_7_) > K_ap_ (Cu_3_(PO_4_)_2_). This kinetic trend strongly correlates with the differences in their active site configurations identified earlier: CuO, which reconstructs into Cu^1+^/Cu^0^ pairs, exhibits superior kinetic activity compared to Cu_2_P_2_O_7_ and Cu_3_(PO_4_)_2_, whose active sites are dominated by Cu^2+^/Cu^0^ pairs. These results provide direct kinetic evidence that the Cu^1+^/Cu^0^ interfacial species possess higher intrinsic activity toward NO_3_^−^ reduction than their Cu^2+^/Cu^0^ pairs.

To confirm the generation of active hydrogen (*H) from water dissociation and its role in the NO_3_RR process, we added tert‐butanol (t‐BuOH), a common active hydrogen quencher,^[^ ^29^ ^]^ to the electrolyte during NO_3_RR testing. As observed in Figure S20a (Supporting Information), both the ammonia yield and FEs of CuO decreased significantly with t‐BuOH addition, directly confirming that quenched *H is a key active species in the ammonia production pathway of the NO_3_RR. Subsequently, the *H generation and enrichment capabilities of the catalyst were evaluated via cyclic voltammetry. As shown in Figure S20b (Supporting Information), the Cu^1+^/Cu^0^ active sites originating from copper oxide exhibited stronger and sharper hydrogen desorption peaks compared to those from Cu_2_P_2_O_7_ and Cu_3_(PO_4_)_2_. This clearly indicates superior *H generation and enrichment capabilities on the Cu^1+^/Cu° catalyst surface.^[^ ^30^ ^]^ Notably, this excellent *H supply characteristic is consistent with the high ammonia yield and high FE during NO_3_RR of the CuO catalyst we observed earlier. This result further confirms that the products formed by the electrochemical reconstruction of Cu‐based catalysts not only provide adsorption and activation sites for NO_3_^−^, but also directly affect the overall catalytic performance of NO_3_RR by regulating the efficiency of *H generation and utilization.

To elucidate the underlying catalytic mechanisms at the Cu^1+^/Cu^0^ and Cu^2+^/Cu^0^ active sites, online differential electrochemical mass spectrometry (DEMS) and in situ Fourier‐transform infrared spectroscopy (FTIR) are employed to track and identify reaction intermediates in real time. These techniques are particularly powerful for capturing transient species, thereby providing critical insights into the reaction pathways. During NO_3_RR, DEMS measurements were conducted by cyclically scanning the potential from ‐0.05 to ‐1.15 V (vs. RHE) over four consecutive cycles, and the mass‐to‐charge ratio (m/z) signals generated at the CuO_re._ and Cu_2_P_2_O_7re._ electrodes are recorded (Figure 4d,e,g,h). The detected major signals corresponded to the following species: NO_2_ (m/z = 46), NOH (m/z = 31), NO (m/z = 30), N (m/z = 14), NH_2_ (m/z = 16), NH_3_ (m/z = 17), and N_2_ (m/z = 28). Further analysis also detected m/z signals corresponding to hydroxylamine (*NH_2_OH), though its signal intensity was notably weak at only 1.5 × 10^−13^ (Figure S21, Supporting Information). It is worth noting that the two key intermediates *NOH and *N (Figure 4d), which exhibited significantly higher signal intensities of 6.8 × 10^−12^ and 5.3 × 10^−11^, respectively. By integrating the temporal evolution patterns of these high‐intensity intermediates (*NOH and *N), the low‐intensity *NH_2_OH signal, and other signals, and drawing upon literature reports on relevant copper‐based catalysts,^[^ ^31^ ^]^ we reasonably summarize the evolution process of the NO_3_RR reconstructed on copper‐based electrodes as follows: *NO_3_^−^→*NO_2_^−^→*NOH→*N→*NH_2_→*NH_3_. This sequence involves sequential deoxygenation and hydrogenation steps: NO_3_^−^ is initial adsorbed and then reduced to *NO_2_^−^, followed by successive deoxygenation steps to form *NO and *NOH; the *NOH intermediate is then converted to a nitrogen‐centered intermediate (*N), which undergoes stepwise hydrogenation to generate *NH_2_ and ultimately *NH_3_. Such a stepwise reduction process optimizes the activation of N‐O bonds and hydrogenation of N species, thereby enhancing the overall reaction efficiency.

Notably, distinct behaviors of intermediate evolution are observed between the two active site configurations. For the Cu^1+^/Cu^0^ pairs (CuO_re._ electrode), the signals of NO_2_ and NO intermediates show a gradual decrease over the four cycles, indicating that nitrogen‐oxygen intermediates are rapidly converted on the electrode surface rather than accumulating. This efficient intermediate turnover facilitates the smooth progression of the reaction toward NH_3_ formation. In contrast, for the Cu^2+^/Cu^0^ pairs (CuPO_re._ electrode), the signals of nitrogen‐oxygen intermediates exhibit a progressive increase over cycles, reflecting sluggish kinetics of intermediate conversion. Importantly, despite these differences in intermediate dynamics, the NH_3_ signals for both active site configurations show a consistent upward trend throughout the cycles, confirming that both systems can drive NO_3_RR toward NH_3_ production, though with varying efficiencies.

To acquire molecular‐level insights into the potential‐dependent evolution of surface‐adsorbed intermediates during NO_3_RR, in situ FTIR spectroscopy is conducted from the OCP to applied potentials ranging from ‐0.45 to ‐1.05 V versus RHE. This approach enabled systematic capture of vibrational signatures of key intermediates on CuO_re._ and Cu_2_P_2_O_7re._ electrocatalysts, providing direct spectral evidence for resolving the reaction pathway. For CuO_re_, which features Cu^1+^/Cu^0^ active sites, a progressive enhancement of the absorption band at 1542 cm^−1^ is observed with increasing cathodic potential (Figure 4f). This band is well‐documented in the literature to correspond to the stretching vibration of *NO_2_ intermediates, and its intensity evolution unequivocally confirms the initial step of *NO_3_^−^→*NO_2_^−^ conversion on the CuO_re._ surface. Concurrently, a set of triplet bands emerged at 1473, 1458, and 1436 cm^−1^, which can be assigned to the bending vibrations (δ(NH_3_)) of *NH_x_ species.^[^ ^31^, ^32^ ^]^ Notably, the intensity of these *NH_3_‐related bands exhibited a clear cathodic‐potential dependence, which strengthen as the potential shifted negatively, and this trend is consistent with the kinetic behavior of NH_3_ formation. For Cu_2_P_2_O_7re._ catalysts containing Cu^2+^/Cu^0^ active sites, the formation of the *NO_2_ intermediate was also detected, indicating that the initial NO_3_^−^ activation step is common to both catalyst systems. Additionally, a distinct absorption band at 1559 cm^−1^ is observed, attributed to the continuous deoxygenation intermediate *NO‐another key species in the NO_3_RR pathway. Furthermore, an absorption band at 1428 cm^−1^ is detected, which is assigned to the bending vibration of the hydrogenated intermediate *NH_2_.^[^ ^30^ ^]^ The gradual intensification of this band with increasing cathodic potential confirms the sequential progression of hydrogenation steps toward the formation of the final NH_3_ product. Collectively, the dynamic correlation and stepwise evolution of these characteristic absorption bands validate the proposed NO_3_RR pathway: *NO_3_ →*NO_2_→*NO→*NH_2_→*NH_3_. This spectroscopic evidence is in excellent agreement with the intermediate evolution trends inferred from DEMS measurements, thereby providing consistent and complementary mechanistic insights into the NO_3_RR process on both Cu^1+^/Cu^0^ and Cu^2+^/Cu^0^ active sites.

**Figure 5:**
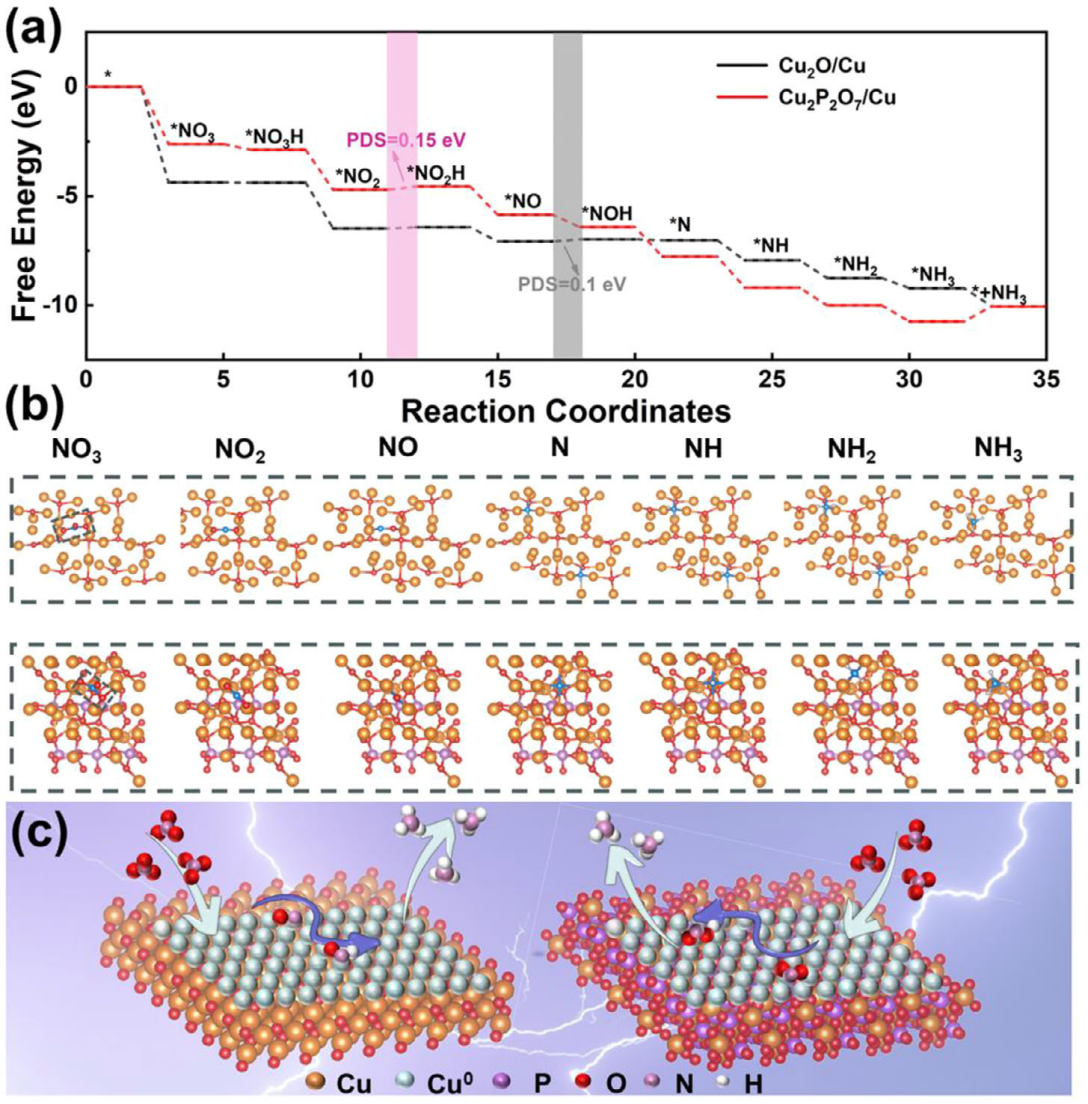
a) Free‐energy diagram for NO_3_RR over Cu_2_O/Cu and Cu_2_P_2_O_7_/Cu; b) Most stable configurations of reaction intermediates on the surface of Cu_2_O/Cu and Cu_2_P_2_O_7_/Cu along the NO_3_RR (color notation: white‐H, pink‐N, red‐O, light purple‐P, yellow‐Cu and bule‐ pure Cu^0^); c) Proposed mechanism of the electrocatalytic NO_3_RR over CuO_re._ and Cu_2_P_2_O_7re._

To further clarify the mechanistic differences in nitrate reduction over Cu^1+^/Cu^0^ and Cu^2+^/Cu^0^ active sites, density functional theory (DFT) calculations are performed. Specifically, computational models are constructed to simulate the Cu^1+^/Cu^0^ active pairs (Cu_2_O/Cu) and the Cu^2+^/Cu^0^ pairs (Cu_2_P_2_O_7_/Cu), with their optimized structural configurations presented in Figure S22 (Supporting Information). Guided by the experimental observations from DEMS and in situ FTIR, the free energy diagrams for NO_3_RR (**Figure** **5**a) and the adsorption configurations of key intermediates (Figure 5b) are subsequently constructed to unravel the reaction pathways. The calculated results reveal the following reaction sequence: Initially, NO_3_^−^ adsorbs onto the electrode surface, where the N─O bonds are spontaneously activated and sequentially cleaved, undergoing a stepwise deoxygenation process to form *NO_3_, *NO_2_, *NO and *N intermediates. For the Cu^2+^/Cu^0^ active pairs (modeled as Cu_2_P_2_O_7_/Cu), the potential‐determining step (PDS) of NO_3_RR is identified as the *NO_2_→*NO_2_H transformation in the initial reaction stage, with a Gibbs free energy change (ΔG) of 0.15 eV (Figure 5a). This value is higher than the free energy barrier for the PDS of the Cu^1+^/Cu^0^ pairs (modeled as Cu_2_O/Cu), which corresponds to the *NO→*NOH step (ΔG = 0.1 eV). This computational finding is consistent with the experimental observations, where rapid consumption and conversion of NO_2_^−^ intermediates are observed over the Cu^1+^/Cu^0^ electrode, supporting the notion that Cu^1+^/Cu^0^ pairs are thermodynamically more favorable for facilitating intermediate conversion. Finally, the *N intermediate undergoes successive protonation to generate NH_3_ as the final product. These results collectively demonstrate that, in comparison to Cu^2+^/Cu^0^ pairs, Cu^1+^/Cu^0^ sites not only lower the overall energy barrier of the NO_3_RR process but also accelerate the consumption and conversion of *NO_2_ intermediates, which is thermodynamically favorable for driving the reaction forward (Figure 5c). This finding thus confirms the superior NO_3_RR activity of Cu^1+^/Cu^0^ sites, with excellent consistency with the experimental observations.

Further investigation of the reaction mechanism of Cu^1+^/Cu^0^, wherein Cu_2_O and Cu were used to characterize the Cu^1+^ and Cu^0^ species on the catalyst surface. LSV measurements on pure Cu^0^ and Cu_2_O (Figure S23, Supporting Information) revealed that catalysts with Cu^1+^/Cu° composite sites exhibit higher current densities, indicating that the high ammonia selectivity originates not from a single site but from the synergy between the two sites. Notably, Cu_2_O shows a higher current density than Cu^0^ at relatively low overpotentials, suggesting that Cu^1+^ sites facilitate the adsorption of NO_3_^−^. Consistent with the LSV results, ammonia yield and FE over Cu_2_O alone were lower than those over the CuO derived Cu^1+^/Cu^0^ sites (Figure S24a,b, Supporting Information). Given the higher yield and FE of NO_2_^−^ over Cu_2_O within the studied potential window (Figure S24c,d, Supporting Information), we reasonably infer that Cu^1+^ sites in the Cu^1+^/Cu^0^ pair promote the conversion of *NO_3_^−^ to *NO_2_^−^. Furthermore, DFT calculations of water dissociation were performed. Figure S25 (Supporting Information) displays the calculated energy profiles for the HER process on Cu_2_O and Cu surfaces. H_2_O first adsorbs on both surfaces, with the dissociation of the *H_2_O species constituting the PDS. The corresponding ΔG value on Cu_2_O is 0.19 eV, lower than the dissociation energy on Cu (ΔG = 0.21 eV), suggesting that *H is more readily generated at the Cu_2_O surface for the NO_3_RR. Subsequently, *OH species desorb from both Cu_2_O and Cu surfaces. The formation of H_2_ from H* is an endothermic reaction, corresponding to the suppression of H‐H coupling and thus inhibiting the HER reaction. The *H dissociated on Cu_2_O sites may readily spill into nearby surface‐exposed metallic Cu^0^ sites for further reduction to NH_3_.^[^ ^33^ ^]^ Thus, Cu^1+^ and Cu^0^ synergistically contribute to the ammonia production via nitrate reduction reaction.

## 3 Conclusion

In summary, we synthesized CuO, Cu_2_P_2_O_7_ and Cu_3_(PO_4_)_2_ (CuPO) pre‐catalysts undergo in situ reconstruction into Cu^1+^/Cu^0^ (CuO‐derived) and Cu^2+^/Cu^0^ (CuPO‐derived) pairs as active species, validated via in situ Raman and XPS characterization. The role of *H species, generated from H_2_O dissociation, in the NO_3_RR process is verified, revealing exceptional *H generation and enrichment capabilities on the Cu^1+^/Cu^0^ active sites. Combined with online DEMS and in situ FT‐IR, the dynamic evolution of reaction intermediates is tracked in real‐time, demonstrating rapid catalytic kinetics of the Cu^1+^/Cu^0^ active pairs for the conversion of *NO_3_ to *NO_2_. Furthermore, DFT calculations unveil the theoretical origin of the optimal reaction energy barrier at the Cu^1+^/Cu^0^ interface. And also confirm that within the Cu^1+^/Cu^0^ active site, Cu^1+^ and Cu^0^ synergistically contribute to the ammonia production via NO_3_RR. The process establishes a crucial theoretical foundation for understanding the catalytic mechanism of the NO_3_RR. Elucidating the origin of the active phase deepens fundamental insights and guides the rational design of high‐performance nitrate‐to‐ammonia electrocatalysts.

## Conflict of Interest

The authors declare no conflict of interest.

## Supporting information



Supporting Information

## Data Availability

The data that support the findings of this study are available from the corresponding author upon reasonable request.
