# Supplementary material for: In Situ Construction of Cu1+/Cu0 and Cu2+/Cu0 Pairs of Cu‐Based Catalysts for Electrocatalytic Nitrate Reduction
Source: Adv Sci (Weinh). 2025 Nov 29;13(9):e17773. doi: 10.1002/advs.202517773 (PMC12904027; doi:10.1002/advs.202517773)
Supplement: Supplementary file 1 — Supporting Information [file ADVS-13-e17773-s001.docx]

**Sopporting Information**

***In-Situ* Construction of Cu^1+^/Cu^0^ and Cu^2+^/Cu^0^ Pairs of Cu-Based Catalysts for Electrocatalytic Nitrate Reduction**

*Shanna An, Jiali Ren, Yanjun Xue, Jian Tian^*^*

*School of Materials Science and Engineering, Shandong University of Science and Technology, Qingdao 266590, China. Email: jiantian@sdust.edu.cn (J. Tian)*

**Experimental section**

**Materials**

Potassium nitrate (KNO_3_) was bought from Chengdu Kelong Chemical Reagent Factory. Copper nitrate trihydrate (Cu(NO_3_)_2_·3H_2_O) was purchased from Shanghai Titan Scientific Co., Ltd. Ammonium dihydrogen phosphate (NH_4_H_2_PO_4_) was obtained from Sinopharm Chemical Reagent Co., Ltd. Citric acid monohydrate (C_6_H_10_O_8_) was purchased from Shanghai Lingfeng Chemical Reagent Co., Ltd. Sodium nitroferricyanide (Ⅲ) dihydrate (Na_2_[Fe(NO)(CN)_5_]·2H_2_O), N-(1-naphthyl) ethylenediamine dihydrochloride (C_12_H_16_C_l2_N_2_), potassium Nitrate-^15^N (K^15^NO_3_, 99 atom%) and p-aminobenzenesulfonamide (C_6_H_8_N_2_O_2_S) and p-dimethylaminobenzaldehyde (p-C_9_H_11_NO) were gained from Shanghai Macklin Biochemical Co., Ltd. Phosphoric acid (H_3_PO_4_, ≥85 wt.%) was purchased from Tianjin Kemiou Chemical Reagent Co., Ltd. Sodium hydroxide (NaOH) was purchased from Yantai Yuandong Fine Biochemical Co., Ltd. Ethanol (C_2_H_5_OH), and sodium sulfate anhydrous (Na_2_SO_4_) was procured from Shanghai Aladdin Biochemical Co., Ltd. Hydrazine hydrate (N_2_H_4_·H_2_O, 85 wt.%), ammonium chloride (NH_4_Cl), sulfuric acid (H_2_SO_4_, 98 wt.%), hydrochloric acid (HCl, 36 wt.%), salicylic acid (C_7_H_6_O_3_), sodium hypochlorite (NaClO, 30 wt.%) and sodium citrate (C_6_H_5_Na_3_O_7_) were purchased from Sinopharm. Nafion solution (D520, 5 wt.%) was acquired from Shanghai Hesen electric Co., Ltd.

**Synthesis of electrocatalysts**

Cu_3_(PO_4_)_2_ was synthesized by a sol-gel method by dissolving 12 mmol Cu(NO_3_)_2_·3H_2_O and 8 mmol NH_4_H_2_PO_4_ in water and stirring thoroughly to form a suspension. Then C_6_H_10_O_8_ was added into it, and stirring was continued until the suspension was clarified. It was then dried in an oven. After drying, it was calcined in a muffle furnace at a heating rate of 5 °C/min for 1 h at 700 °C. Cu_2_P_2_O_7_ was prepared in the same way as the above procedure except that the mass of Cu(NO_3_)_2_·3H_2_O was changed to 8.5 mmol. Cu was prepared in the same way as that of Cu_3_(PO_4_)_2_ except that NH_4_H_2_PO_4_ was not added.

**Characterization**

The crystal structures was identified by X-ray diffraction (XRD) performed on a Rigaku Ultima IV diffractometer (Japan), with Cu Kα radiation source. The morphology, component and structure of samples were examined by scanning electron microscope (SEM) on FEI Nova Nano SEM 450 (USA) and transmission electron microscope (TEM) equipped with Energy Dispersive Spectrometer (EDS) system on JEOL JEM2100F (Japan) with an accelerating voltage of 200 kV. The chemical states of samples were analyzed by X-ray photoelectron (XPS) performed on Thermo ESCALAB 250XI (USA) with the C 1s binding energy of 284.8 eV. UV-vis absorption spectra were collected by UV-2600i ultraviolet-visible spectrophotometer of SHIMADZU (Japan) to analyze these electrolytic products after NO_3_RR process. ^1^H nuclear magnetic resonance (NMR) spectra were also collected to quantify the yield of NH_3_ product recorded on a Bruker FT-NMR AVANCE III HD 600MHz spectrometer.

**Electrochemical measurements**

The catalysts were dissolved in a water-ethanol solution (1:1 volume ratio, 960 µL). Subsequently, 20 µL of Nafion suspension (5 wt%) was added into the above solution, and the solution was thoroughly mixed by ultrasonic treatment. Following this, the catalyst suspension (20 µL) was dropped onto the glassy carbon electrode with a diameter of 6 mm and then dried at room temperature. All electrochemical measurements were performed on a CHI 660E electrochemical workstation. Electrocatalysis reaction took place in a conventional H-type three-electrode system, which was divided into a cathode chamber and an anode chamber by a Nafion 117 membrane. The prepared catalysts, Pt mesh, and saturated Ag/AgCl were used as working, counter, and reference electrodes, respectively. All potentials in this paper were converted to reversible hydrogen electrodes (RHE) by this equation: E (*vs.* RHE) = E (*vs.* Ag/AgCl) + 0.197 + 0.0591 × pH. Linear sweep voltammetry (LSV) tests were conducted to roughly assess NO_3_RR performance in 0.1 M Na_2_SO_4_ with 0.1 M KNO_3_. Chronoamperometry tests were carried out in 0.1 M Na_2_SO_4_ with 0.1 M KNO_3_. Various products after electrolysis (NH_4_^+^, NO_3_^-^, N_2_H_4_ and NO_2_^-^) were detected using colorimetric method. Electrochemical impedance spectroscopy (EIS) measurements were performed in the frequency range of 0.1 Hz to 100 kHz at a sinusoidal voltage amplitude of 5 mV.

**Electrochemical active surface areas (ECSAs) measurements**

Electrochemical active surface areas (ECSAs) were measured in the non-Faradaic region from -0.11 V to -0.21 V by cyclic voltammetry (CV) with different scanning rates (60~140 mV s^-1^) in 0.1 M NaSO_4_ with 0.1 M KNO_3_. Double-layer capacitances (*C*_dl_) can be easily obtained by plotting the current density as a function of scan rate in CV curves. And the *C*_dl_ value is proportional to ECSA, meaning a higher *C*_dl_ represents a larger ECSA for these studied catalysts.

**Determination of ammonia by UV-vis method**

The concentration of NH_3_ produced was determined by indophenol blue spectrophotometry. In detail, 2 mL aliquot of the solution was taken out of the cathode chamber of the H-type electrolytic cell. Then, add 2 mL of 1.0 M NaOH solution containing 5 wt.% salicylic acid and 5 wt.% sodium citrate, followed by the addition of 1 mL of 0.05 M NaClO and 0.2 mL of an aqueous solution of 1 wt.% Na_2_[Fe(NO)(CN)_5_] 2H_2_O. After 2 h at room temperature, the UV-vis absorption spectrum was tested using a UV-2600i ultraviolet-visible spectrophotometer. The absorbance at a wavelength of 655 nm was used to determine the formation of indoxyl blue. Using standard NH_4_Cl solutions with a range of concentrations to calibrate the concentration-absorbance curve.

**Determination of hydrazine**

A mixture of p-(dimethylamino)benzalde hyde (5.99 g), 1.0 M HCl (30 mL) and ethanol (300 mL) was used as a color reagent. 5mL of electrolyte solution was extracted from the electrolytic cell, and then 5mL of color reagent was added and mixed uniformly. After the mixture solution was let stand for 10 min, the absorption intensity at a wavelength of 456 nm was recorded.

**Determination of nitrite**

A mixture of p-aminobenzenesulfonamide (4 g), N-(1-Naphthyl) ethylenediamine dihydrochloride (0.2 g), ultrapure water (100 mL) and phosphoric acid (10 mL, ρ=1.70 g/mL) was used as a color reagent. 1.0 mL of the electrolyte was removed from the electrolytic cell and diluted to 5 mL to the detection range. Next, 0.1 mL of the color reagent was added into the aforementioned 5 mL solution and mixed uniformly. After the mixture solution was let stand for 20 min, the absorption intensity at a wavelength of 540 nm was recorded. The concentration-absorbance curve was calibrated using a series of standard sodium nitrite solutions.

**Isotope labelling experiment**

100 μL deuterium oxide (D_2_O) was added in 0.55 mL above mixed solution for the NMR test. The calibration can be acquired using the peak area ratio between ^15^NH_4_^+^-^15^N and DMSO against the concentration of ^15^NH_4_^+^-^15^N. Similarly, the calibration curve of ^14^NH_4_^+^-^14^N can be also obtained by the above method. As for the quantification of generated ammonia via nitrate electroreduction, K^15^NO_3_ with 99 atom % isotope abundance and K^14^NO_3_ were used as the reactants, respectively. All the quantifications of ammonia were through the ^1^H-NMR.

**Calculation of the yield rate, and Faradaic efficiency**

The yield rate of electrocatalytic NO_3_^−^-NH_3_ was defined according to Eq. (1):

${}_{\mathrm{NH}_{3}}=(C_{\mathrm{NH}_{3}}V)/(tm_{cat.})$ (1)

The content of the by-product NO_2_^−^ is calculated similarly, except that C_NH3_ is replaced by C_NO2-_ The Faradaic efficiency of the electrocatalytic NO_3_^−^-NH_3_ was defined from the electric charge consumed for synthesizing ammonia and total charge passed through the electrode according to Eq. (2):

$\mathrm{FE}_{\mathrm{NH}_{3}}=(8FC_{\mathrm{NH}_{3}}V)/(17Q)$ (2)

The Faradaic efficiency of electrocatalytic NO_3_^−^–NO_2_^−^ was defined from the electric charge consumed for synthesizing ammonia and total charge passed through the electrode according to Eq. (3):

${FE}_{{NO}_{2}^{-}}=(2FC_{{NO}_{2}^{-}}V)/(46Q)$ (3)

where C_NH3_ is the concentration of NH_3_(aq), V is the volume of electrolyte in the cathode compartment (70 mL), t is the electrolysis time (2 h), C is the generated concentration of ammonia or nitrite, F is the Faradaic constant (96485 C mol^-1^), Q is the total charge passing the electrode, and m_cat._ is the loading mass of catalysts.

**DFT calculations**

DFT calculations were carried out using the generalized gradient approximation (GGA) with Perdew−Burke−Ernzerhof (PBE)^[1]^ functional to treat the electron interactions. The projector-augmented wave (PAW)^[2]^ method was used to describe the core and valence electrons. The energy cutoff was set at 400 eV for all calculations. The energy and force convergence thresholds were set to be 1×10^-5^ eV and 0.02 eV Å^-1^, respectively. The free energy change (ΔG) for adsorptions were determined as follows:

$\Delta G=E_{total}-E_{slab}-E_{mol}+\Delta E_{ZPE}-T\Delta S$ (4)

Where E_total_ is the total energy for the adsorption state, E_slab_ is the energy of pure surface, E_mol_ is the energy of adsorption molecule, ΔE_ZPE_ is the zero-point energy change and ΔS is the entropy change.


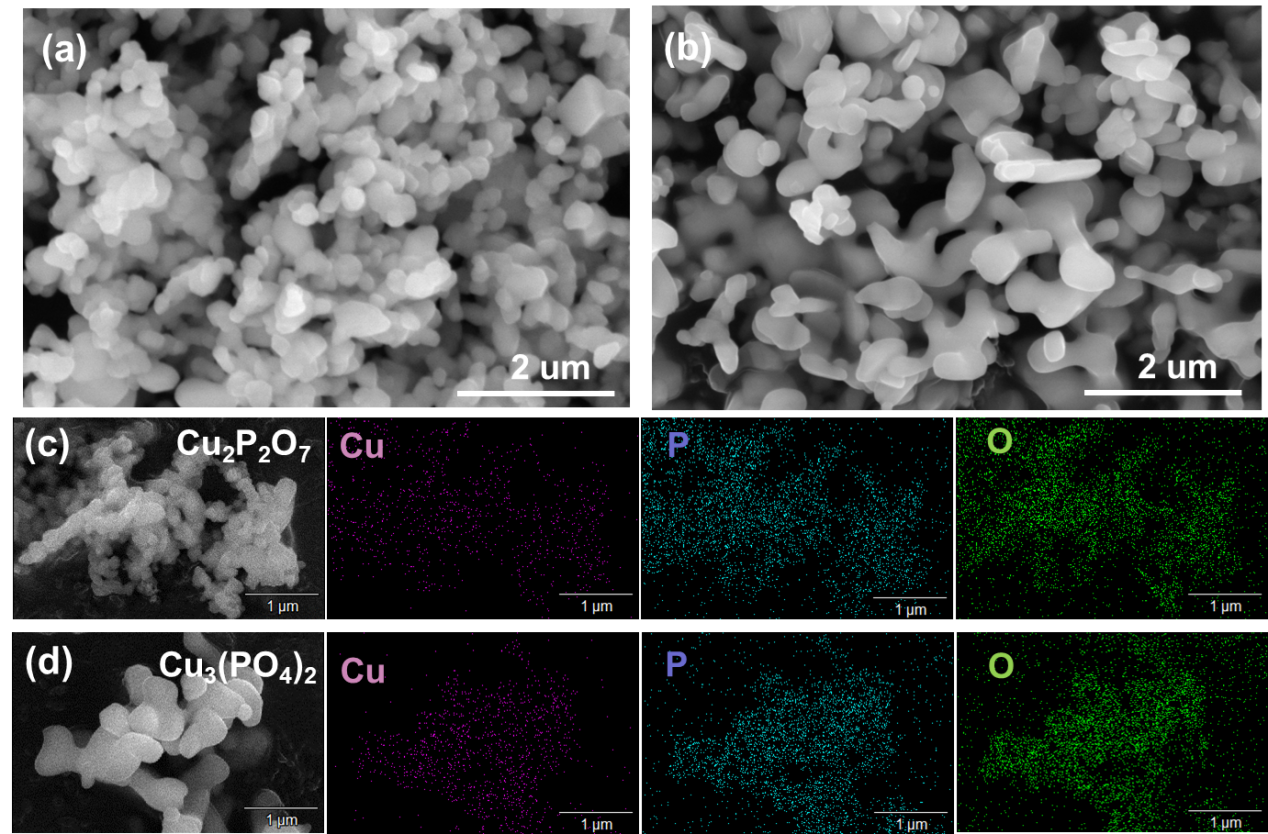


**Figure S1.** SEM images of (a) Cu_2_P_2_O_7_ and (b) Cu_3_(PO_4_)_2_; SEM-EDS images of (c) Cu_2_P_2_O_7_ and (d) Cu_3_(PO_4_)_2_.


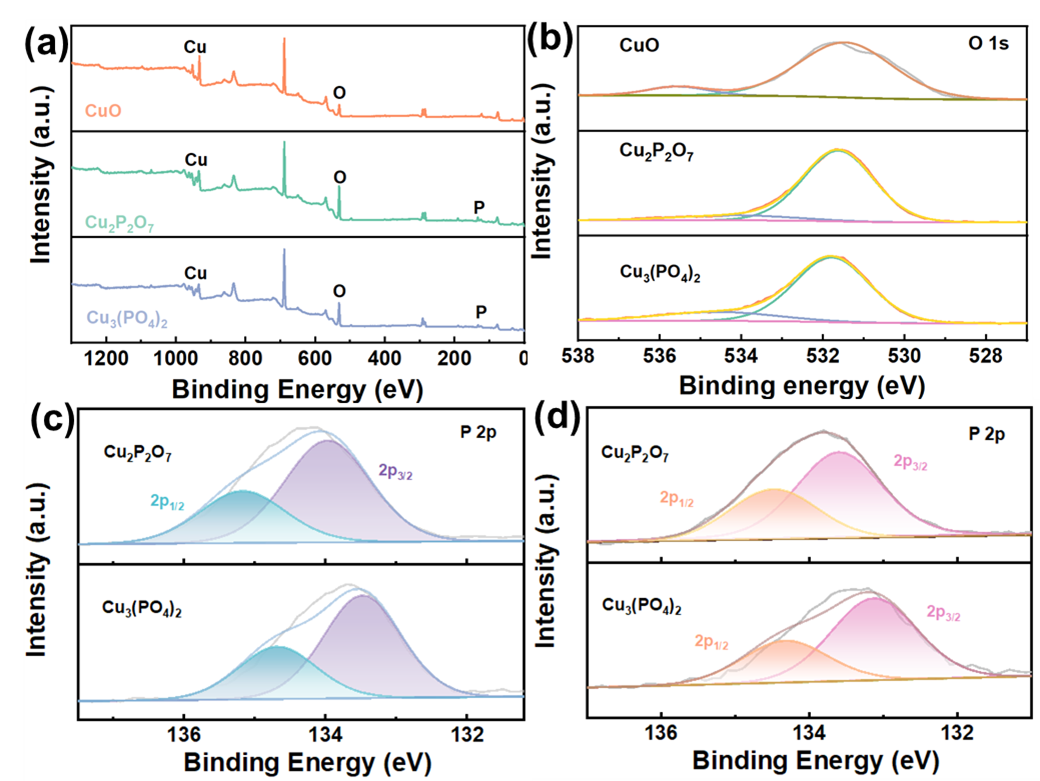


**Figure S2.** (a) Survey and (b) O 1s XPS spectra of CuO, Cu_2_P_2_O_7_, and Cu_3_(PO_4_)_2_; P 2p XPS spectra of Cu_2_P_2_O_7_, and Cu_3_(PO_4_)_2_ (c) before and (c) after reaction.


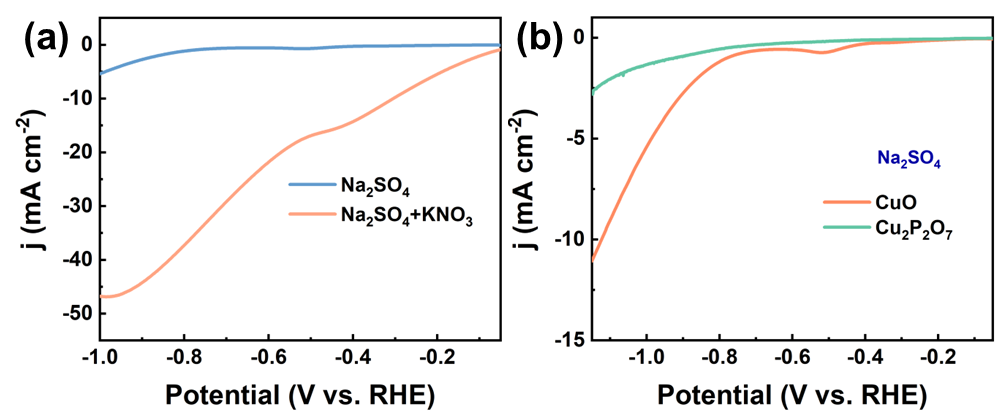


**Figure S3.** LSV curves of (a) CuO measured on glassy carbon electrodes in 0.1 M Na_2_SO_4_ with or without 1.0 M NO_3_^–^; (b) CuO and Cu_2_P_2_O_7_ in 0.1 M Na_2_SO_4_.


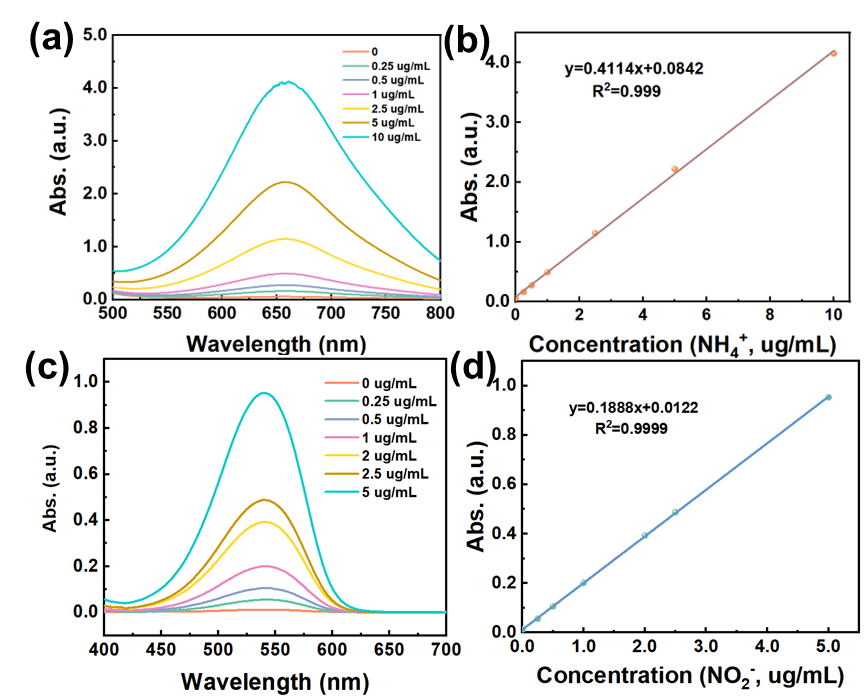


**Figure S4.** (a, b) UV-vis absorption spectra and calibration curves of ^14^NH_4_^+^; (c, d) UV-vis absorption spectra and calibration curves of NO_2_^−^.


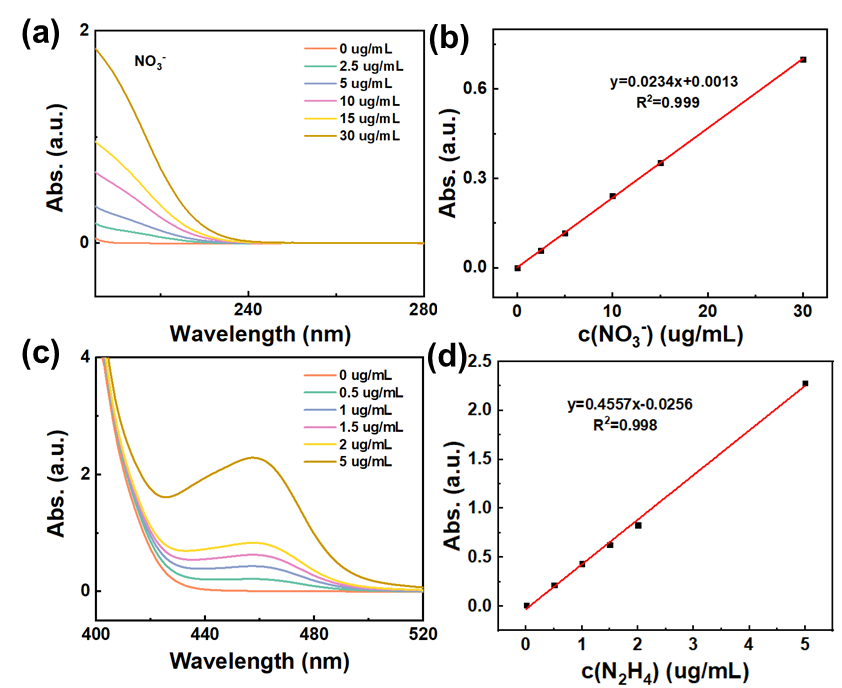


**Figure S5.** (a, b) UV-vis absorption spectra and calibration curves of NO_3_^-^; (c, d) UV-vis absorption spectra and calibration curves of N_2_H_4_.


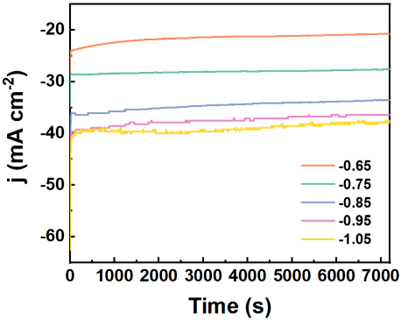


**Figure S6.** Chronoamperometry tests of CuO at different potentials in 0.1 M NO_3_^-^ containing electrolyte.


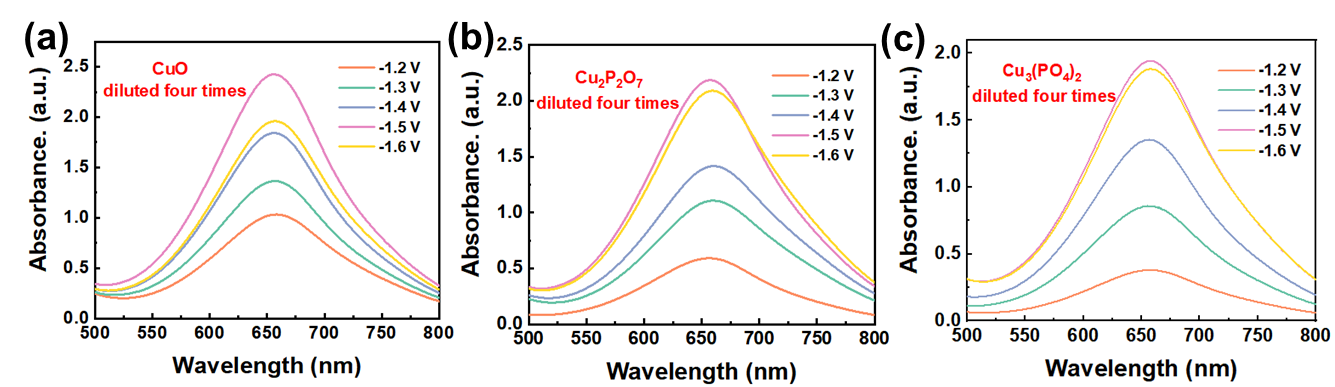


**Figure S7.** UV- vis absorption spectra of post-electrolysis electrolyte of (a) CuO, (b) Cu_2_P_2_O_7_ and (c) Cu_3_(PO_4_)_2_.


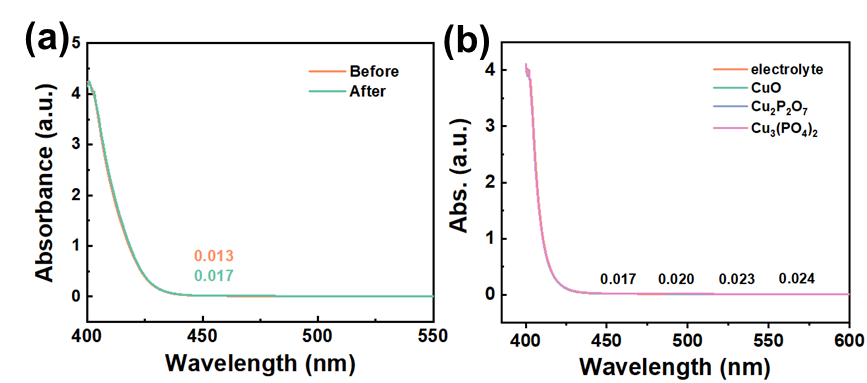


**Figure S8.** UV-vis absorption spectra of N_2_H_4_·H_2_O (a) before and after 2h NO_3_RR tested at -0.95 V of electrolyte, (b) after 2h NO_3_RR tested at -0.95 V.


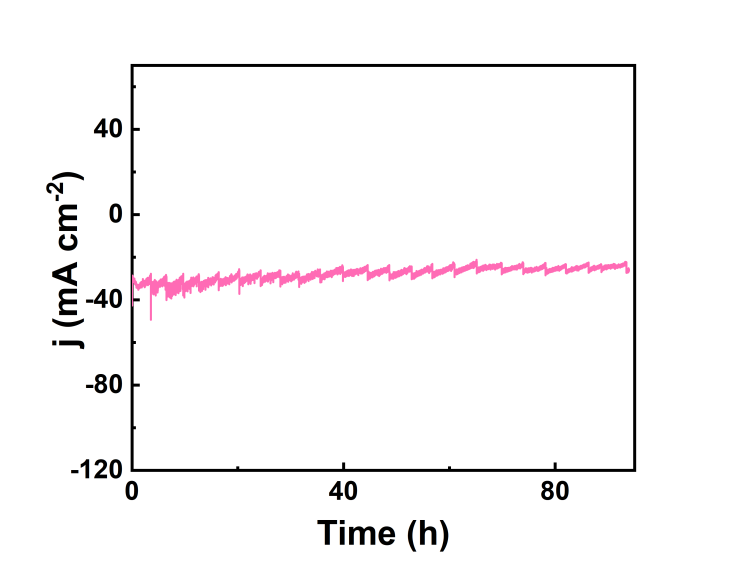


**Figure S9.** 95 h long-time I-t curves of CuO.


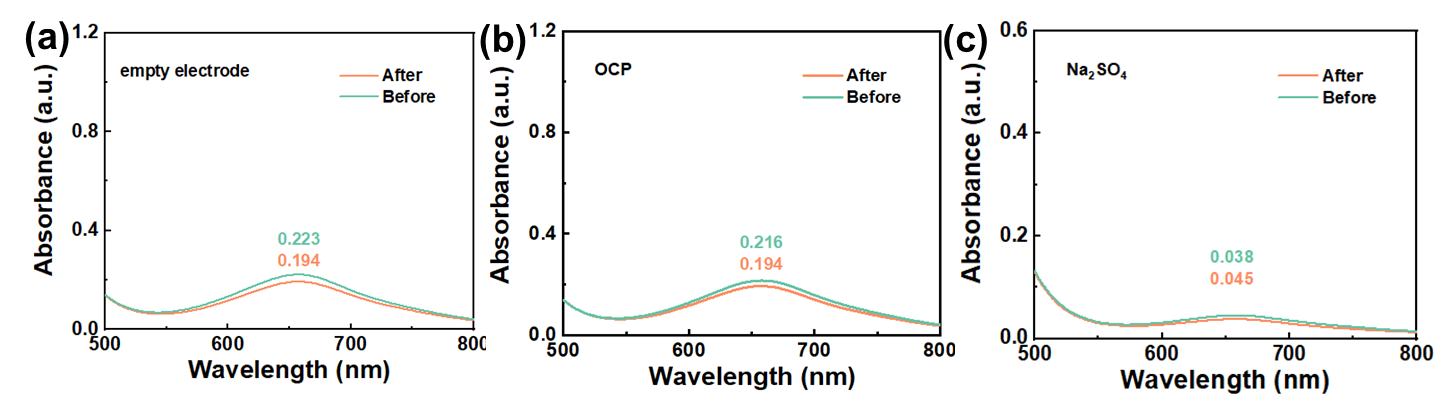


**Figure S10.** (a) UV-vis absorption spectra of post-electrolysis solution (a) with empty electrode, (b) at an open circuit potential in 0.1 M Na_2_SO_4_/0.1 M KNO_3_, and (c) in pure 0.1 M Na_2_SO_4_ solution before and after NO_3_RR for 2 h, respectively.


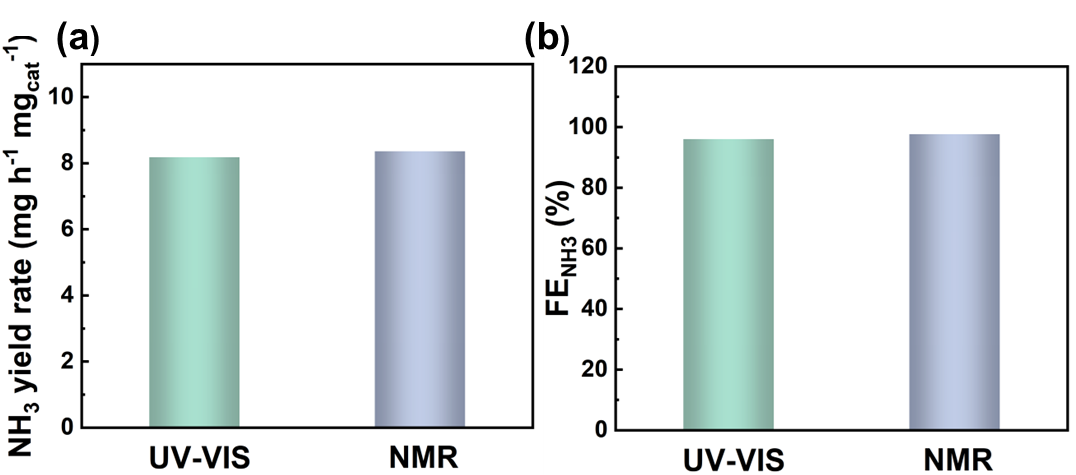


**Figure S11.** Comparison of the measured NH_3_ yield rate and FE over CuO after electrocatalytic NO_3_RR at -0.95 V (vs. RHE) based on the UV-VIS and 1H NMR methods.


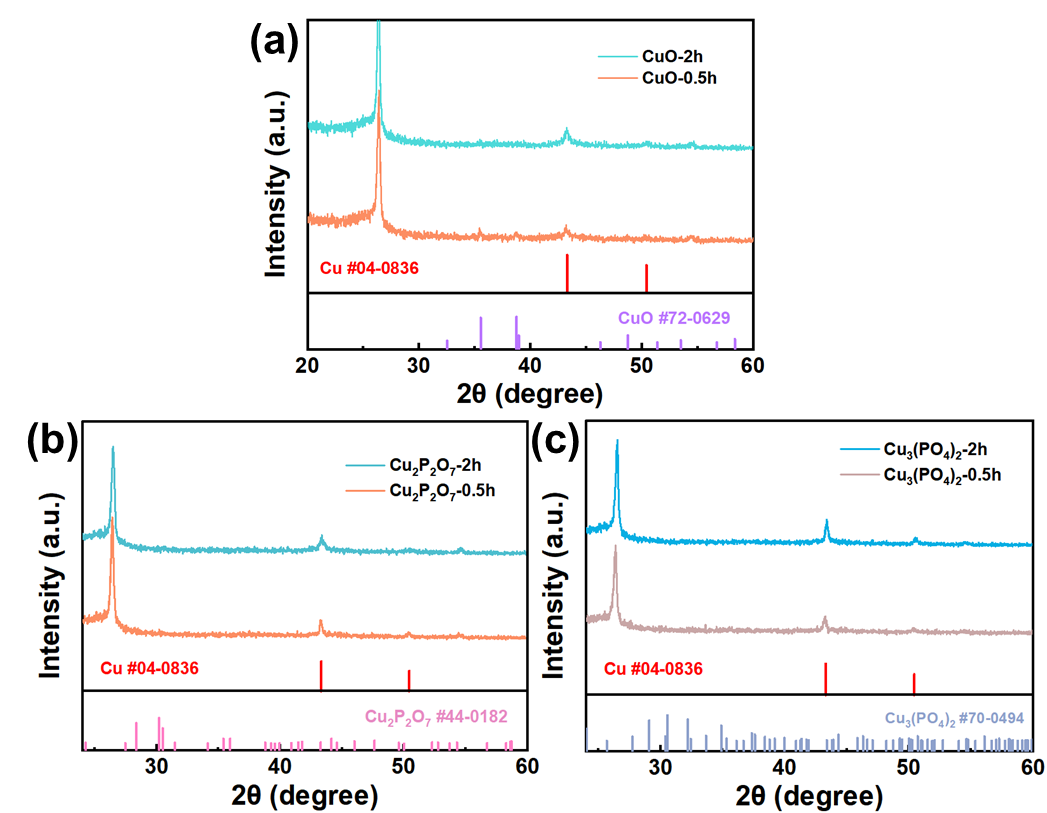


**Figure S12.** XRD patterns of (a) CuO, (b) Cu_2_P_2_O_7_ and (c) Cu_3_(PO_4_)_2_ loaded on carbon paper with NO_3_RR reaction for 0.5h and 2h.


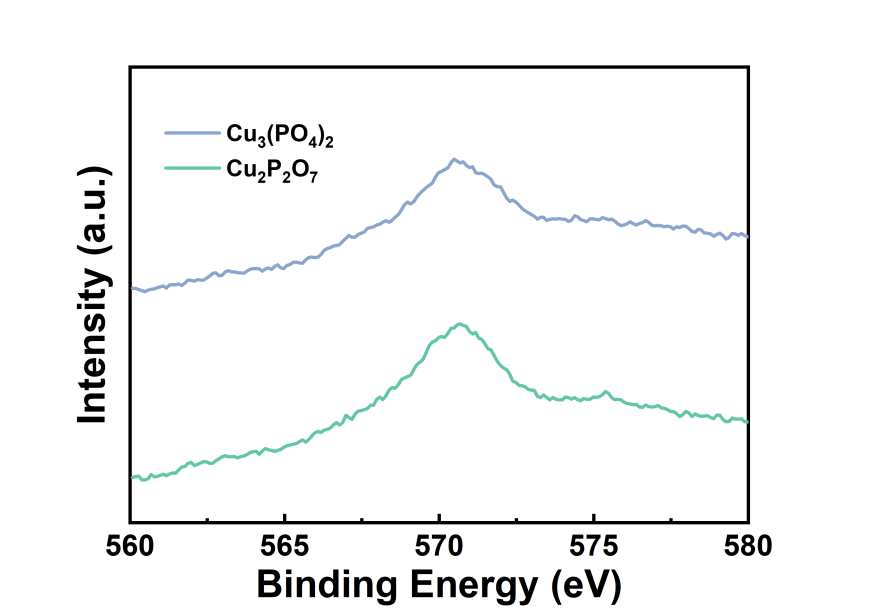


**Figure S13.** Cu LMM AES spectra of (a) Cu_2_P_2_O_7_ and Cu_3_(PO_4_)_2_.


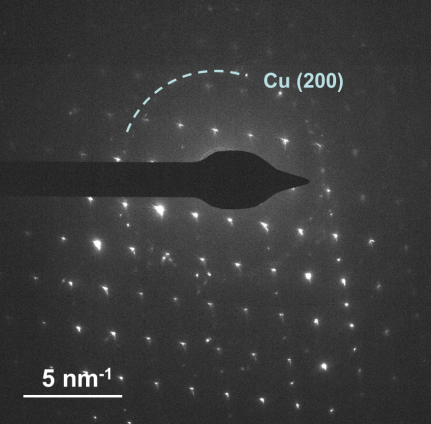


**Figure S14.** SAED image of Cu_3_(PO_4_)_2re._.


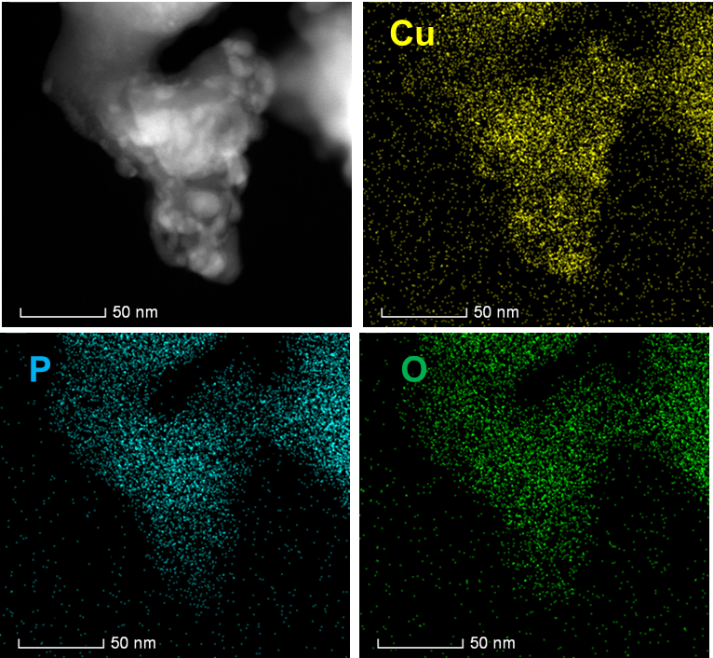


**Figure S15.** Enlarged HAADF-STEM and corresponding EDS elemental mapping images of Cu_3_(PO_4_)_2re._.


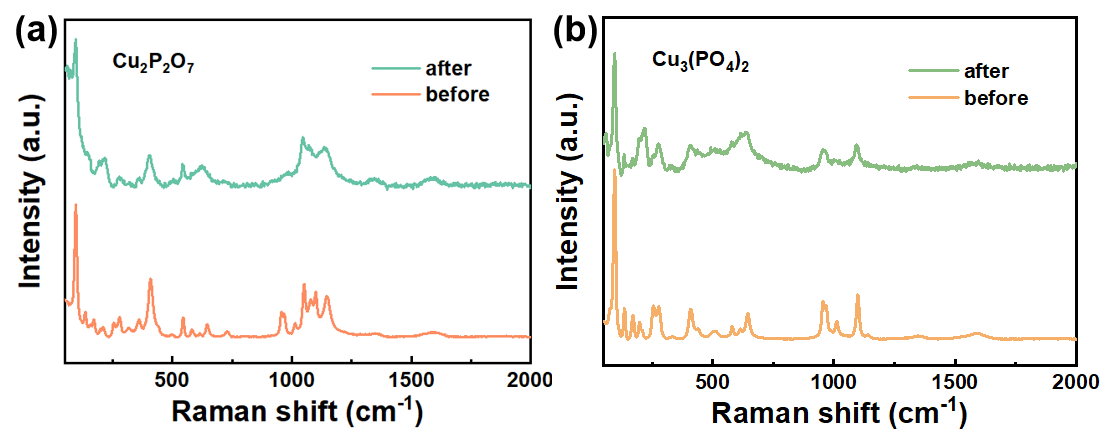


**Figure S16.** Raman spectra of (a) Cu_2_P_2_O_7_ and (b) Cu_3_(PO_4_)_2_ before and after the reaction loading on carbon paper.


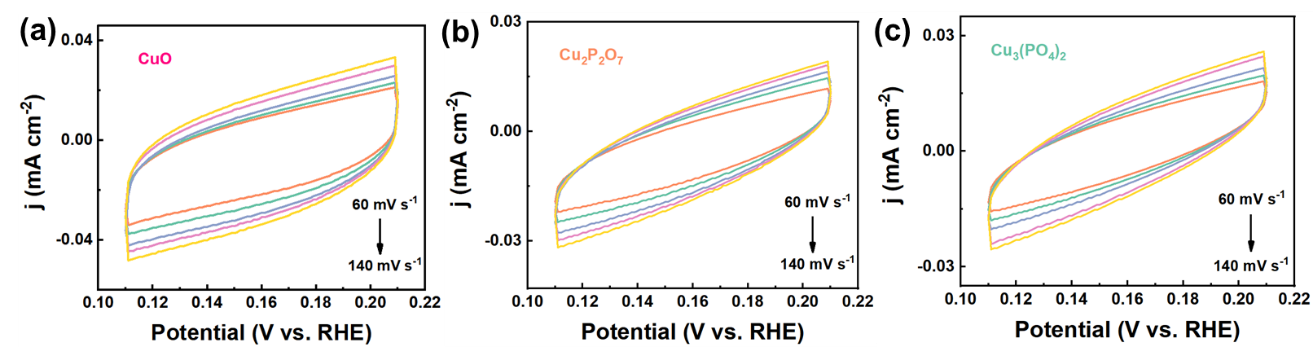


**Figure S17.** CV curves of (a) CuO, (b) Cu_2_P_2_O_7_, and (c) Cu_3_(PO_4_)_2_ at different scan rates from 60 to 140 mV s^-1^ in the non-Faradic region.


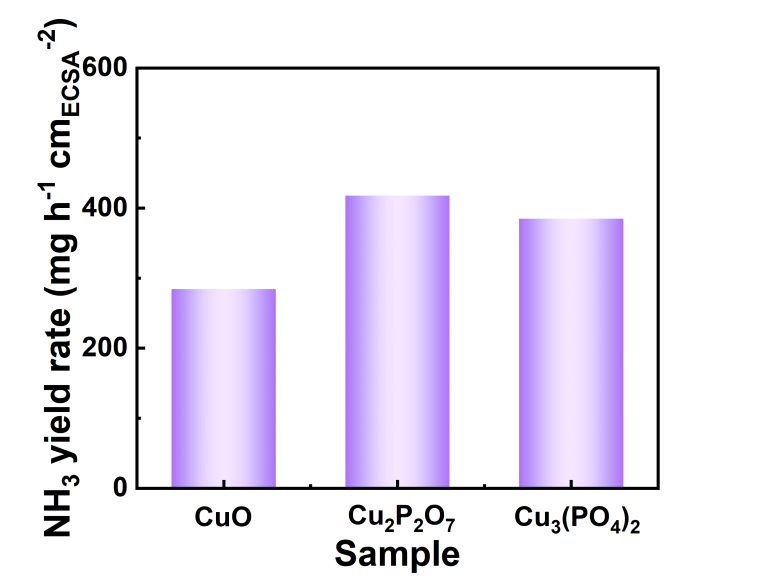


**Figure S18.** Ammonia production rates of different samples standardized by ECSA at -0.95 V *vs.* RHE. The specific capacitance for a flat surface is generally found to be in the range of 20-60 µF cm^-2^. In the following calculations of electrochemical active surface area, we assume 40 µF cm^-2^ (Cs). $A_{\mathrm{ECSA}}=(C_{\mathrm{dl}})/(C_{s.})$.


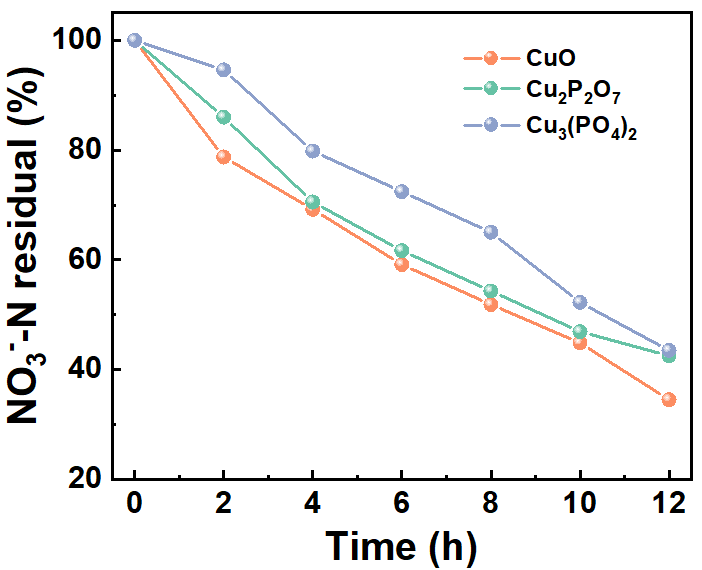


**Figure S19.** Change in percentage of nitrate in electrolyte.


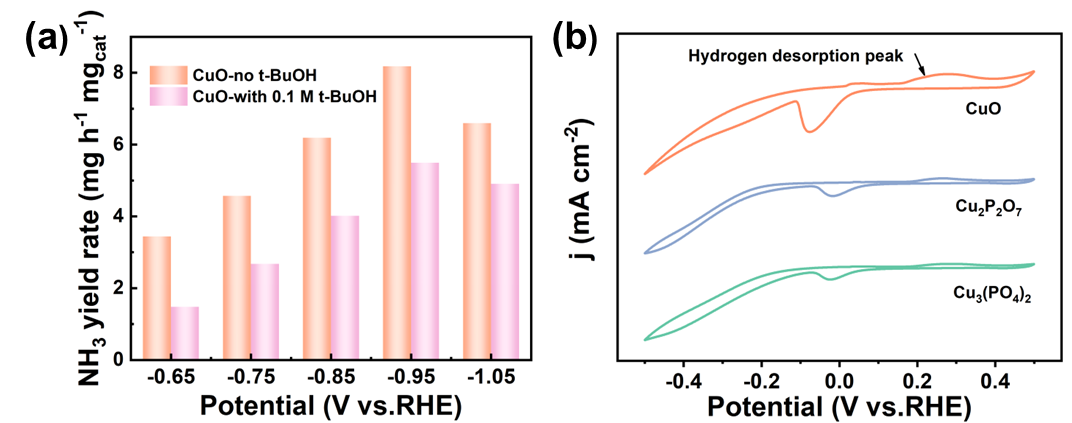
**Figure S20.** (a) NH_3_ yield rate of CuO at different potentials in 0.1M Na_2_SO_4_+0.1 M KNO_3_ with and without 0.1M t-BuOH; (b) CV curves of the samples in 0.1M Na_2_SO_4_+0.1 M KNO_3_.


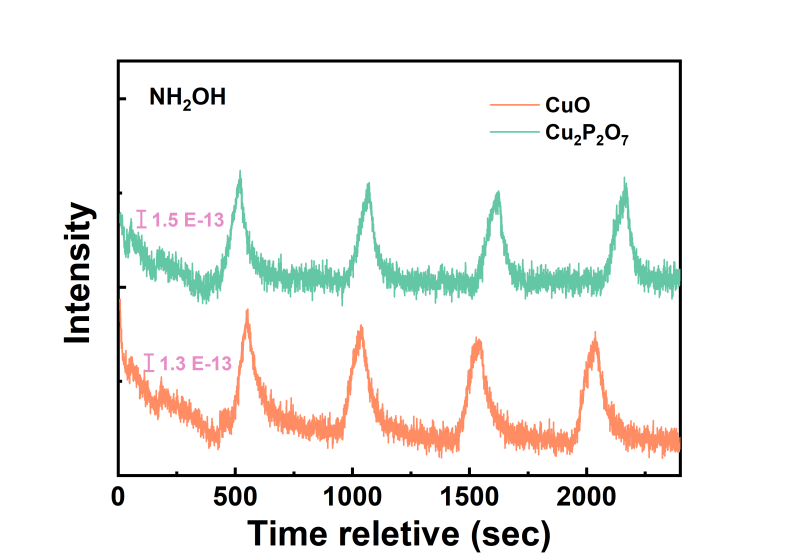


**Figure S21.** Electrochemical online DEMS results (NH_2_OH) for the electrocatalytic NO_3_RR over CuO_re._.


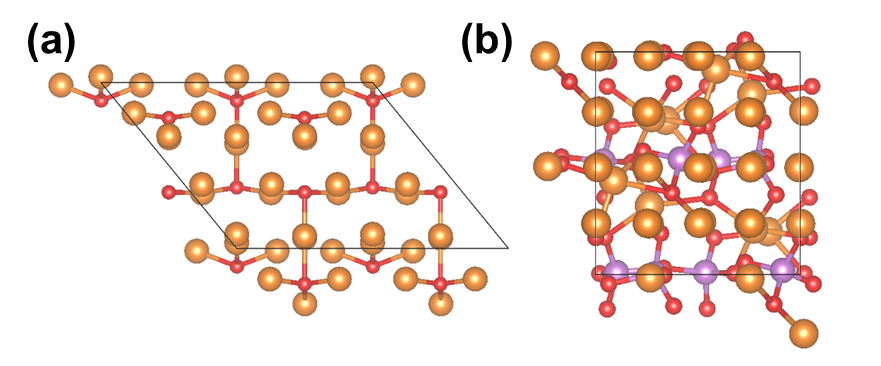


**Figure S22.** Local crystal structure of (a) Cu_2_O/Cu and (b) Cu_2_P_2_O_7_/Cu.


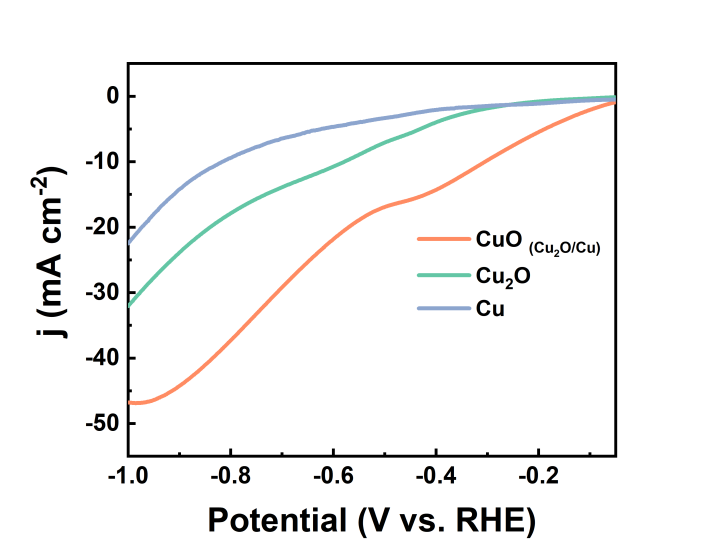


**Figure S23** LSV curves of Cu, Cu_2_O and CuO (derived Cu^1+^/Cu^0^) in 0.1 M N_2_SO_4_ with 0.1 M KNO_3_.


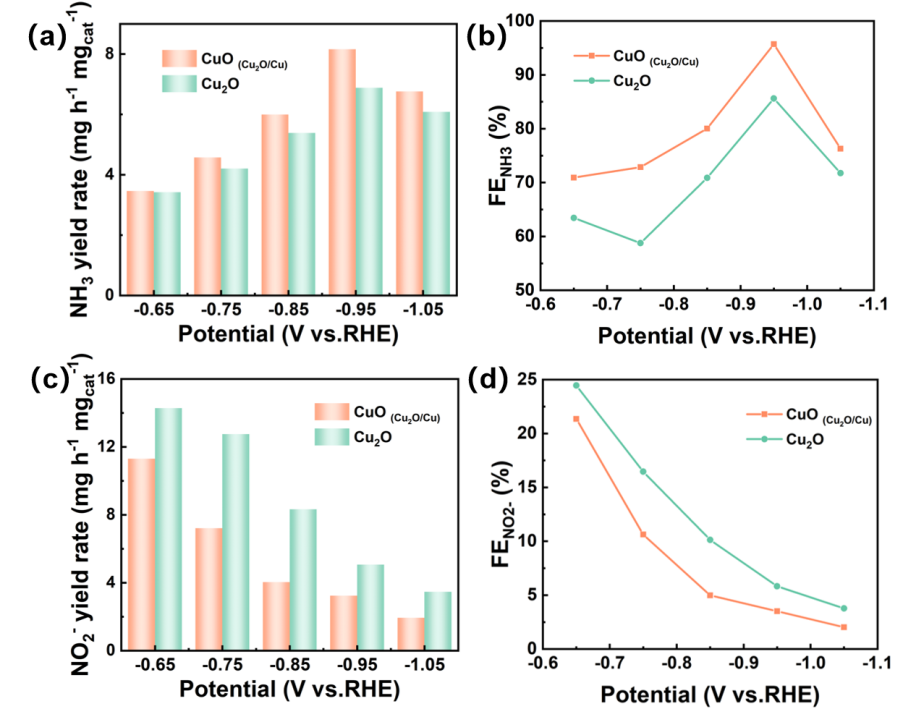


**Figure S24** (a) NH_3_ yield rate and (b) Faradaic efficiency and (c) NO_2_^-^ yield rate and (d) Faradaic efficiency of CuO (derived Cu^1+^/Cu^0^) and Cu_2_O at different potentials.


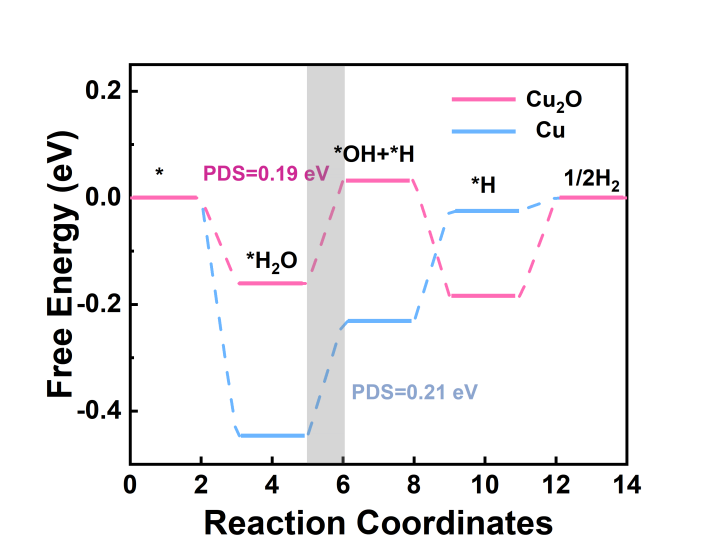


**Figure S25.** Free-energy diagram for HER over Cu_2_O and Cu.

**Table S1.** A summary of recently developed electrocatalysts for the NO_3_RR at ambient temperatures.

| Catalyst | NH_3_ yield rate | FE (%) | Electrolyte | Electrode | Ref |
| --- | --- | --- | --- | --- | --- |
| CuO | 8.16 mg h^-1^ mg^-1^ | 95.72 | 0.1 M Na_2_SO_4_+0.1M KNO_3_ | glassy carbon disk | This work |
| Cu_2_P_2_O_7_ | 7.33 mg h^-1^ mg^-1^ | 94.88 | 0.1 M Na_2_SO_4_+0.1M KNO_3_ | glassy carbon disk | This work |
| Cu_3_(PO_4_)_2_ | 6.53 mg h^-1^ mg^-1^ | 92.04 | 0.1 M Na_2_SO_4_+0.1M KNO_3_ | glassy carbon disk | This work |
| Ru_SACs_-CF | 2326 µg h^-1^ cm^-2^ | 82.2 | 0.2 M Na_2_SO_4_+1000 mg L^-1^ N-NO_3_- | Ru_SACs_-CF | [3] |
| Cu-N-C | 4500 µg h^-1^ cm^-2^ | 84.7 | 0.1 M KOH+0.1M N-NO_3_- | GDL substrate | [4] |
| Fe/Cu-HNG | 2300 µg h^-1^ cm^-2^ | 58 | 1 M KOH+0.1M N-NO_3_- | carbon paper | [5] |
| Ru_1_Cu_10_/rGO | 1.3 mg h^-1^ cm^-2^ | 63 | 1 M KOH+0.1M KNO_3_ | carbon felt | [6] |
| Cu_30%_@NHC | 3628.28 µg h^-1^ mg^-1^ | 85.9 | 0.1 M Na_2_SO_4_+0.1M KNO_3_ | Cu_30%_@NHC | [7] |
| Fe_2_Co-MOF | 3463 µg h^-1^ mg^-1^ | 90.55 | 0.05 M H_2_SO_4_ (50 g/L NO_3_^-^) | carbon paper | [8] |
| S5-Co_3_O_4_ | 2960 µg h^-1^ mg^-1^ | 89.9 | 0.1 M Na_2_SO_4_+0.1 M NaNO_3_ | carbon cloth | [9] |
| Fe-NC | 4330.9 µg h^-1^ mg^-1^ | 80 | 1 M KOH+0.1 M KNO_3_ | / | [10] |
| Plasma Cu_2_O | 1402.5 µg h^-1^ mg^-1^ | 89.54 | 0.5 M Na_2_SO_4_+50  ppm NaNO_3_ | carbon paper | [11] |
| Cu SAGs | 0.44 mg h^-1^ cm^-2^ | 78.0 | 0.1 M PBS + 0.02 M NO_3_ | carbon paper | [12] |
| NiO_4_-CCP | 2.15 mg h^-1^ cm^-2^ | 94.7 | 1 M Na_2_SO_4_ + 0.5 M KNO_3_ | carbon paper | [13] |
| Co-B@CoOx | 0.056 mmol h^-1^ cm^-2^ | 86.82 | 0.5 M Na_2_SO_4_+100ppm NO_3_^-^ | Ni foam | [14] |
| CuFe DS/NC | 112.52 µmol h^-1^ cm^-2^ | 90 | 0.1 mol/L Na_2_SO_4_ (NO) | carbon paper | [15] |
| Fe-N/P-C | 5.8 mg h^-1^ mg^-1^ | 90.3 | 0.1 M KOH + 0.1 M KNO_3_ | carbon paper | [16] |
| a-RuO_2_ | 1.97 mg h^-1^ cm^-2^ | 97.4 | 0.5 M Na_2_SO_4_ + 200 ppm NO_3_^-^ | carbon paper | [17] |
| meso-PdN NCs | 3.76 mg h^-1^ mg^-1^ | 96.1 | 0.1 M Na_2_SO_4_ + 0.5 M KNO_3_ | carbon paper | [18] |
| LaSrNiCoMnFeCuO_3_ PNTs | 1.66 mg h^-1^ mg^-1^ | 100 | 0.5 M K_2_SO_4_+KNO_3_ (500 mg L^-1^) | glassy carbon electrode | [19] |
| Co+Bi@Cu NW | 1.86 mg h^-1^ cm^-2^ | 99.51 | 0.1 M Na_2_SO_4_+50 mM NaNO_3_ | / | [20] |
| i-Ag/Co_3_O_4_ NWs | 253.7 µmol h^-1^ cm^-2^ | 94.3 | 1 M KOH + 0.1 M KNO_3_ | carbon paper | [21] |
| NiPr-TPA-COF | 147.1 µmol h^-1^ cm^-2^ | 90 | 0.5 M K_2_SO_4_ + 0.1 M NO_3_^-^ | carbon fiber paper | [22] |
| Ag-Co_3_O_4_ | 0.884 mg h^-1^ cm^-2^ | 88 | 0.1 M KOH +  0.1 M KNO_3_ | carbon fiber paper | [23] |

**References**

[1] J.P. Perdew, K. Burke, M. Ernzerhof, *Phys. Rev. Lett.* **1996**, 77, 3865-3868.

[2] P.E. Blöchl, *Phys. Rev. B Condens. Matter.* **1994**, 50, 17953-17979.

[3] T. Xiang, X. Liu, Z. Wang, Y. Zeng, J. Deng, W. Xiong, M. Cheng, J. Liu, C. Zhou, Y. Yang, *Appl. Catal. B-Environ. Energy* **2025**, 365, 124943.

[4] J. Yang, H. Qi, A. Li, X. Liu, X. Yang, S. Zhang, Q. Zhao, Q. Jiang, Y. Su, L. Zhang, J.-F. Li, Z.-Q. Tian, W. Liu, A. Wang, T. Zhang, *J. Am. Chem. Soc.* **2022**, 144 (27), 12062.

[5] S. Zhang, J. Wu, M. Zheng, X. Jin, Z. Shen, Z. Li, Y. Wang, Q. Wang, X. Wang, H. Wei, J. Zhang, P. Wang, S. Zhang, L. Yu, L. Dong, Q. Zhu, H. Zhang, J. Lu, *Nat. Commun.* **2023**, 14 (1), 3634.

[6] Gao, W.; Xie, K.; Xie, J.; Wang, X.; Zhang, H.; Chen, S.; Wang, H.; Li, Z.; Li, C, *Adv. Mater.* **2023**, 35 (19), 2202952.

[7] J. Zhang, C. Chen, R. Zhang, X. Wang, Y. Wei, M. Sun, Z. Liu, R. Ge, M. Ma, J. Tian, *J. Colloid Interf. Sci*. **2024**, 658, 934-942.

[8] Y. Lv, S.-W. Ke, Y. Gu, B. Tian, L. Tang, P. Ran, Y. Zhao, J. Ma, J.-L. Zuo, M. Ding, *Angew. Chem., Int. Ed.* **2023**, 62 (27), e202305246.

[9] Z. Niu, S. Fan, X. Li, J. Yang, J. Wang, Y. Tao, G. Chen, *Chem. Eng. J.* **2023**, 451, 138890.

[10] J. Wan, J. Yang, N. Yang, Y. Sun, C. Hu, Y. Zhao, X. Xu, H. Qi, X. Li, H. Zhang, *ACS Catal.* **2025**, 15(6), 4507-4518.

[11] Z. Gong, W. Zhong, Z. He, Q. Liu, H. Chen, D. Zhou, N. Zhang, X. Kang, Y. Chen, *Appl. Catal. B-Environ. Energy* **2022**, 305, 121021.

[12] P. Li, R. Li, Y. Liu, M. Xie, Z. Jin, G. Yu, J. Am. Chem. Soc. **2023**, 145, 6471-6479.

[13] Y. Zhang, H. Zheng, K. Zhou, J. Ye, K. Chu, Z. Zhou, L. Zhang, T. Liu, Adv. Mater. **2023**, 35, 2209855.

[14] [X. Zhu](https://pubs.rsc.org/en/results?searchtext=Author:Xiaojuan%20Zhu), [C. Ma](https://pubs.rsc.org/en/results?searchtext=Author:Chaoqun%20Ma), [Y. Wang](https://pubs.rsc.org/en/results?searchtext=Author:Yi-Chi%20Wang), [K. Qu](https://pubs.rsc.org/en/results?searchtext=Author:Kaiyu%20Qu), [L. Song](https://pubs.rsc.org/en/results?searchtext=Author:Leyang%20Song), [J. Wang](https://pubs.rsc.org/en/results?searchtext=Author:Jing%20Wang), [Y. Gong](https://pubs.rsc.org/en/results?searchtext=Author:Yushuang%20Gong), [X. Liu](https://pubs.rsc.org/en/results?searchtext=Author:Xiang%20Liu), [Jintao Zhang](https://pubs.rsc.org/en/results?searchtext=Author:Jintao%20Zhang), [Q. Lu](https://pubs.rsc.org/en/results?searchtext=Author:Qipeng%20Lu), [A. Wang](https://pubs.rsc.org/en/results?searchtext=Author:An-Liang%20Wang), Energy Environ. Sci., **2024**, 17, 2908.

[15] [D. Wang](https://advanced.onlinelibrary.wiley.com/authored-by/Wang/Dongdong), [X. Zhu](https://advanced.onlinelibrary.wiley.com/authored-by/Zhu/Xiaorong), [X. Tu](https://advanced.onlinelibrary.wiley.com/authored-by/Tu/Xiaojin), [X. Zhang](https://advanced.onlinelibrary.wiley.com/authored-by/Zhang/Xiaoran), [C. Chen](https://advanced.onlinelibrary.wiley.com/authored-by/Chen/Chen), [X. Wei](https://advanced.onlinelibrary.wiley.com/authored-by/Wei/Xiaoxiao), [Y. Li](https://advanced.onlinelibrary.wiley.com/authored-by/Li/Yafei), [S. Wang](https://advanced.onlinelibrary.wiley.com/authored-by/Wang/Shuangyin), Adv. Mater. **2023**, 35, 2304646.

[16] J. Xu, S. Zhang, H. Liu, S. Liu, Y. Yuan, Y. Meng, M. Wang, C. Shen, Q. Peng, J. Chen, X. Wang, L. Song, K. Li, W. Chen, Angew. Chem. Int. Ed. **2023**, 62 (39), e202308044.

[17] Y. Wang, H. Li, W. Zhou, X. Zhang, B. Zhang, Y. Yu, Angew. Chem. Int. Ed. **2022**, 61 (19), e202202604.

[18] L. Sun, B. Liu, Adv. Mater. **2023**, 35 (1), 2207305.

[19] Y. Chen, C. Chen, W. Huang, C. Pao, C. Chang, T. Mao, J. Wang, H. Fu, F. Lai, N. Zhang, T. Liu, ACS Nano. **2024**, 18, 20530-20540.

[20] [R. Zhao](https://advanced.onlinelibrary.wiley.com/authored-by/Zhao/Rundong), [Q. Yan](https://advanced.onlinelibrary.wiley.com/authored-by/Yan/Qiuyu), [L. Yu](https://advanced.onlinelibrary.wiley.com/authored-by/Yu/Lihong), [T. Yan](https://advanced.onlinelibrary.wiley.com/authored-by/Yan/Tian), [X. Zhu](https://advanced.onlinelibrary.wiley.com/authored-by/Zhu/Xuya), [Z. Zhao](https://advanced.onlinelibrary.wiley.com/authored-by/Zhao/Zongyan), [L. Liu](https://advanced.onlinelibrary.wiley.com/authored-by/Liu/Le), [J. Xi](https://advanced.onlinelibrary.wiley.com/authored-by/Xi/Jingyu), Adv. Mater. **2023,** 35, 2306633.

[21] S. Wu, Y. Jiang, W. Luo, P. Xu, L. Huang, Y. Du, H. Wang, X. Zhou, Y. Ge, J. Qian, H. Nie, Z. Yang, Adv. Sci. **2023**, 10, 2303789.

[22] F. Lv, M. Sun, Y. Hu, J. Xu, W. Huang, N. Han, B. Huang, Y. Li, Energy Environ. Sci. **2023**, 16, 201-209.

[23] M. Zhang, Z. Ma, S. Zhou, C. Han, V. Kundi, P. V. Kumar, L. Thomsen, B. Johannessen, L. Peng, Y. Shan, C. Tsounis, Y. Yang, J. Pan, R. Amal, ACS Catal. **2024**, 14, 11231-11242.
